# Prognostic impact of chromosomal abnormalities and copy number alterations in adult B-cell precursor acute lymphoblastic leukaemia: a UKALL14 study

**DOI:** 10.1038/s41375-021-01448-2

**Published:** 2021-10-16

**Authors:** Anthony V. Moorman, Emilio Barretta, Ellie R. Butler, Eleanor J. Ward, Katie Twentyman, Amy A. Kirkwood, Amir Enshaei, Claire Schwab, Tom Creasey, Daniel Leongamornlert, Elli Papaemmanuil, Pip Patrick, Laura Clifton-Hadley, Bela Patel, Tobias Menne, Andrew K. McMillan, Christine J. Harrison, Clare J. Rowntree, David I. Marks, Adele K. Fielding

**Affiliations:** 1 Leukaemia Research Cytogenetics Group, Translational and Clinical Research Institute, Newcastle University, Newcastle upon Tyne, UK; 2 Cancer Research UK & UCL Cancer Trials Centre, UCL Cancer Institute, University College London, London, UK; 3 Sanger Institute, Cambridge, UK; 4 Memorial Sloan Kettering Cancer Center, New York, NY USA; 5 Department of Haematology, Queen Mary University of London, London, UK; 6 Department of Haematology, Newcastle upon Tyne Hospitals NHS Foundation Trust, Newcastle upon Tyne, UK; 7 Department of Haematology, Nottingham University Hospital NHS Trust, Nottingham, UK; 8 Department of Haematology, Cardiff And Vale University Health Board, Cardiff, UK; 9 Department of Haematology, University Hospitals Bristol NHS Foundation Trust, Bristol, UK; 10 UCL Cancer Institute, London, UK

**Keywords:** Cancer genetics, Risk factors, Acute lymphocytic leukaemia, Cytogenetics

## Abstract

Chromosomal abnormalities are established prognostic markers in adult ALL. We assessed the prognostic impact of established chromosomal abnormalities and key copy number alterations (CNA) among 652 patients with B-cell precursor ALL treated on a modern MRD driven protocol. Patients with *KMT2A-AFF1*, complex karyotype (CK) and low hypodiploidy/near-triploidy (HoTr) had high relapse rates 50%, 60% & 53% and correspondingly poor survival. Patients with *BCR-ABL1* had an outcome similar to other patients. JAK-STAT abnormalities (*CRLF2*, *JAK2*) occurred in 6% patients and were associated with a high relapse rate (56%). Patients with ABL-class fusions were rare (1%). A small group of patients with *ZNF384* fusions (*n* = 12) had very good survival. CNA affecting *IKZF1, CDKN2A/B, PAX5, BTG1, ETV6, EBF1, RB1* and PAR1 were assessed in 436 patients. None of the individual deletions or profiles were associated with survival, either in the cohort overall or within key subgroups. Collectively these data indicate that primary genetic abnormalities are stronger prognostic markers than secondary deletions. We propose a revised UKALL genetic risk classification based on key established chromosomal abnormalities: (1) very high risk: CK, HoTr or JAK-STAT abnormalities; (2) high risk: *KMT2A* fusions; (3) Tyrosine kinase activating: *BCR-ABL1* and ABL-class fusions; (4) standard risk: all other patients.

## Introduction

The outcome for adults with acute lymphoblastic leukaemia (ALL) treated with multi-agent chemotherapy remains unsatisfactory. Adoption of treatment elements from paediatric protocols, targeted therapy for specific subtypes, improved disease monitoring and better risk stratification have benefited some patient subgroups [1]. However, further advances are required especially for older adults for whom treatment intensification is not feasible due to toxicity [1]. Accurate stratification according to the risk of treatment failure is vital for effective patient management and improving outcome. A profusion of genomic studies over the past 10–15 years has identified numerous recurrent genetic abnormalities. The prognostic or predictive value of these potential biomarkers has yet to be fully determined in adult ALL.

The UKALLXII/ECOG2993 international trial generated one of the first widely used risk classifiers in adult ALL, showing that patients harbouring *BCR-ABL1*, *KMT2A-AFF1*, low hypodiploidy/near triploidy (HoTr), or complex karyotype (CK) had an inferior survival [2–4]. This study identified the presence of minimal residual disease (MRD) at the end of induction and age >40 years as risk factors for poor outcome [5, 6]. These findings were applied in UKALL14 and patients with one or more of these risk features were assigned to the high-risk arm and allocated to allogeneic stem cell transplant (allo-SCT) wherever possible [7].

Retrospective studies examining the prognostic impact of copy number alterations (CNA) or mutations have reported correlations with outcome but the magnitude of the effect was often marginal, frequently restricted to specific genetic subtypes and rarely replicated across independent cohorts [8–18]. Hence, the majority of stratification algorithms in adult ALL focus on primary chromosomal abnormalities rather than secondary CNA or mutations, although there are rare exceptions [11]. Genomic studies have identified and characterised large numbers of additional primary genetic abnormalities, including ABL-class fusions and abnormalities leading to deregulated JAK-STAT signalling, which underpin the BCR-ABL1-like gene expression signatures [19–21]. Initial studies suggested the frequency of BCR-ABL1-like ALL increased with age and was associated with a poor outcome [22–26]. Moreover, there are emerging data to suggest that patients with an ABL-class fusion benefit from targeted therapy with a tyrosine kinase inhibitor [27–29].

In this study, we sought to assess the prognostic impact of the most relevant primary and secondary genetic abnormalities in a large cohort of patients aged 25–65 years with ALL treated on a single clinical trial, UKALL14.

## Materials and methods

Full details of patients and methods are provided in the supplementary methods section. Briefly patients (25-65 years) were enroled onto UKALL14 (ISRCTN 66541317), a phase 3 clinical trial for patients with *de novo* ALL between 2010 and 2018. All patients provided written informed consent to trial treatment and correlative science studies. Patients underwent a two-phase induction followed by stratification to continuing chemotherapy or allogeneic stem cell transplant (allo-SCT) based on risk assessment. Patients were assigned to high-risk treatment if they had any of the following features: *BCR-ABL1, KMT2A-AFF1*, HoTr, CK, WCC > 30 × 10^9^/l, MRD post phase 2 induction and ≥41 years. High-risk patients were assigned to allo-SCT if they were fit and had an antigen-matched sibling or unrelated donor.

Cytogenetics, FISH, MLPA and SNP array analyses were performed on diagnostic bone marrow samples by regional NHS genetic laboratories or the Leukaemia Research Cytogenetics Group at Newcastle University as previously described [8, 30]. (Supplementary Methods, Fig. S1, Table S1/S2) Principal genetic abnormalities were defined as *BCR-ABL1*/t(9;22)(q34;q11), *KMT2A-AFF1*/t(4;11)(q21;q23), other *KMT2A* fusions (together referred to as *KMT2A*-r), HoTr (30–39/60–78 chromosomes), CK (≥5 chromosomal abnormalities), JAK-STAT abnormalities (*CRLF2 or JAK2* fusions), ABL-class fusions (*ABL1, ABL2, PDGFRB, CSF1R* fusions, except *BCR-ABL1*), *ETV6-RUNX1*, high hyperdiploidy (51–65 chromosomes), *ZNF384* fusions (*ZNF384*-r), and t(1;19)(q21;p13)/*TCF3-PBX1*. MRD analysis was preformed and interpreted by the UK Adult ALL MRD lab according to EuroMRD guidelines [31]. MRD was measured after both phases of induction but only the post-phase 2 results were used to assign treatment. Survival analysis focussed on event-free survival (EFS), relapse rate (RR) and overall survival (OS) at 3 years which were calculated using Kaplan–Meier methods (see supplementary methods). Hazard ratios were estimated using univariable and multivariable Cox regression models. All *P* values were two-sided and, because of multiple testing, values <0.01 were considered statistically significant. All analyses were performed using Intercooled Stata v16 (StataCorp, College Station, TX) and R version 3.4.3 (http://www.R-project.org).

## Results

### Frequency and key clinical correlates of primary chromosomal abnormalities

**Fig. 1:**
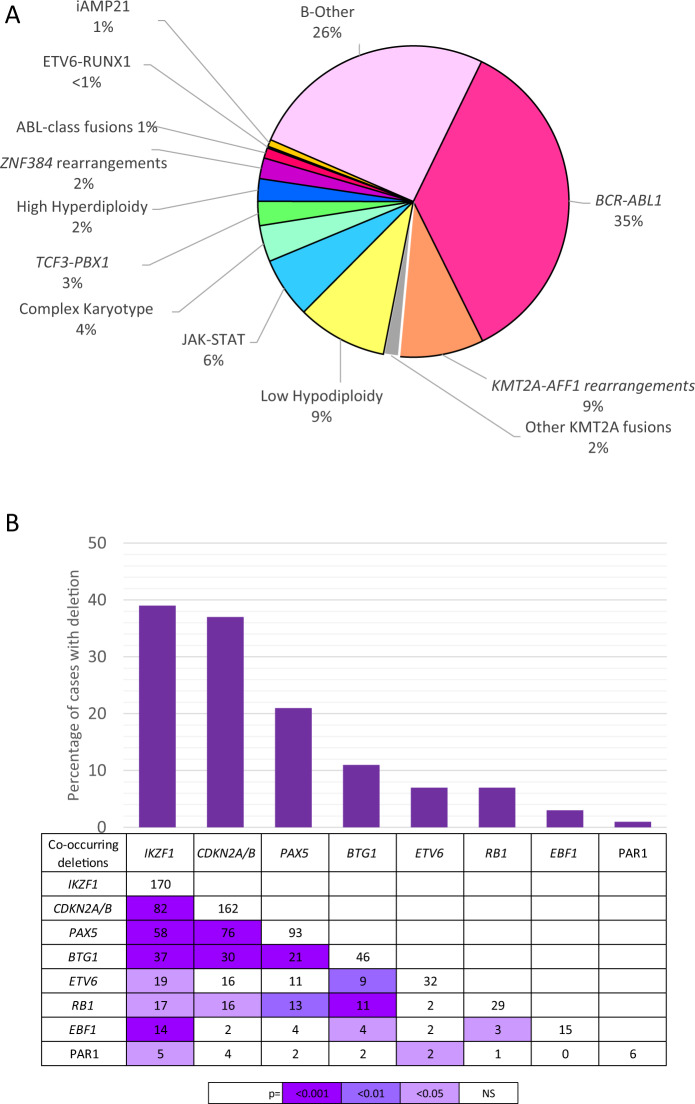
Frequency of primary chromosome abnormalities (A) and secondary copy numbers alterations (B) in adults with B-cell precursor ALL enroled to UKALL14. **A** Pie chart showing the frequency of chromosomal abnormalities. Most *KMT2A* rearranged cases had *KMT2A-AFF1* (i.e., t(4;11), 49/58, 84%) but other partner genes were observed: *MLLT1* (*n* = 6) and *MLLT4*, *EPS15*, *LASP1*, unknown (one each). The low hypodiploidy (30–39 chromosomes) group included two cases of near-haploidy (26–28 chromosomes). The JAK-STAT group comprised *IGH-CRLF2* (*n* = 23), *P2RY8-CRLF2* (*n* = 9) and *JAK2* fusions (*n* = 2, *PAX5-JAK2* & *BCR-JAK2*). *P2RY8-CRLF2* fusions were identified by MLPA (*n* = 4), FISH (*n* = 3) or both techniques (*n* = 2). We were able to identify the partner gene in 3/6 cases: *EBF1-PDGFRB* (*n* = 2), *FOXP1-ABL1* (*n* = 1). The remaining three cases had a *PDGFRB/CSF1R* (*n* = 2) or *ABL1* (*n* = 1) rearrangement. Twelve patients had a *ZNF384* rearrangement with *EP300* (*n* = 5) and *TCF3* (*n* = 4) being the most prevalent partners along with single cases of *AKAP8-ZNF384* and *EWSR1-ZNF384* and one where the partner gene is unknown. **B** Bar chart and table showing the frequency and co-occurrence of copy number alterations.

A total of 652 patients with B-cell precursor ALL were included in this study (Table 1, Fig. S1). The median age of the cohort was 46 years with an interquartile range of 35–55 years. *BCR-ABL1* was the most prevalent abnormality (31%) followed by *KMT2A-r* and HoTr (10% each) (Table 1, Fig. 1). Overall, 319 (49%) patients’ disease harboured a pre-defined high-risk genetic abnormality (*BCR-ABL1*, *KMT2A-AFF1*, HoTr, CK). Most *KMT2A-*r cases had *KMT2A-AFF1* (49/58, 84%) but other partner genes were observed: *MLLT1* (*n* = 6) and *MLLT4*, *EPS15*, *LASP1*, unknown (one each). Conventional karyotype revealed a CK in 21 (4%) cases. None of the CK cases harboured *BCR-ABL1*, *KMT2A-r* or *TCF3-PBX1* and the majority (>80%) were also negative for ABL-class, JAK-STAT and *ZNF384-r*.

**Table 1:** Demographic characteristics, clinical features and outcome of UKALL14 patients according to immunophenotype and principal genetic abnormalities.

	Total cohort	Principal genetic subtype^1^										
		*BCR-ABL1*	*KMT2A-AFF1*	*KMT2A* other	Low Hypodiploid^2^	JAK-STAT abnormalities^3^	Complex Karyotype	*TCF3-PBX1*	High Hyperdiploid	*ZNF384* fusion^4^	ABL-class^5^	B-other^6^
	*N* (%)	*N* (%)	*N* (%)	*N* (%)	*N* (%)	*N* (%)	*N* (%)	*N* (%)	*N* (%)	*N* (%)	*N* (%)	*N* (%)
Total	652	197	49	9	52	35	21	14	13	12	6	148
Frequency^7^	–	35%	8%	2%	10%	6%	4%	3%	3%	2%	1.3%	27%
Sex												
Male	358 (55)	110 (56)	23 (47)	3 (33)	26 (50)	23 (66)	9 (43)	7 (50)	9 (69)	9 (75)	3 (50)	85 (57)
Female	294 (45)	87 (44)	26 (53)	6 (67)	26 (50)	12 (34)	12 (57)	7 (50)	4 (31)	3 (25)	3 (50)	63 (57)
Age												
<40	213 (33)	56 (28)	18 (37)	1 (11)	7 (13)	14 (40)	6 (29)	8 (57)	7 (54)	8 (67)	4 (67)	52 (35)
≥40	439 (67)	141 (72)	31 (63)	8 (89)	45 (87)	21 (60)	15 (71)	6 (43)	6 (46)	4 (33)	2 (33)	96 (65)
White cell count (x10^9^/L)												
<30 × 10^9^/L	484 (74)	124 (63)	7 (14)	4 (44)	50 (96)	26 (74)	18 (86)	10 (71)	11 (85)	9 (75)	5 (83)	132 (89)
≥30 × 10^9^/L	168 (26)	73 (37)	42 (86)	5 (56)	2 (4)	9 (26)	3 (14)	4 (29)	2 (15)	3 (25)	1 (17)	16 (11)
MRD status post-phase 1^8^												
Positive	253 (62)	106 (68)	24 (71)	1 (100)	16 (64)	21 (88)	9 (75)	0 (0)	3 (38)	7 (58)	2 (50)	43 (52)
Negative	158 (38)	51 (32)	10 (29)	0 (0)	9 (36)	3 (13)	3 (25)	8 (100)	5 (63)	5 (42)	2 (50)	40 (48)
MRD status post-phase 2^8^												
Positive	133 (37)	54 (39)	15 (56)	1 (100)	7 (32)	16 (76)	5 (45)	1 (8)	2 (33)	5 (45)	1 (25)	21 (29)
Negative	228 (63)	84 (61)	12 (44)	0 (0)	15 (68)	5 (24)	6 (55)	11 (92)	4 (67)	6 (55)	3 (75)	51 (71)
UKALL14 trial risk group												
Standard risk	87 (13)	0 (0)	0 (0)	0 (0)	0 (0)	6 (17)	0 (0)	6 (43)	6 (46)	3 (25)	2 (33)	37 (25)
High risk	565 (87)	197 (100)	49 (100)	9 (100)	52 (100)	29 (83)	21 (100)	8 (57)	7 (54)	9 (75)	4 (67)	111 (75)
Post-induction treatment^9^												
Chemotherapy	127 (19)	17 (9)	6 (12)	0 (0)	4 (8)	8 (23)	5 (24)	6 (43)	6 (46)	6 (50)	1 (17)	42 (28)
Myeloablative allo-SCT	107 (16)	45 (23)	9 (18)	1 (11)	6 (12)	5 (14)	3 (14)	2 (14)	1 (8)	3 (25)	3 (50)	16 (11)
Reduced intensity allo-SCT	232 (36)	70 (36)	15 (31)	4 (44)	20 (38)	11 (31)	7 (33)	4 (29)	5 (38)	2 (17)	1 (17)	56 (38)
Other	186 (29)	65 (33)	19 (39)	4 (44)	22 (42)	11 (31)	6 (29)	2 (14)	1 (8)	1 (8)	1 (17)	34 (23)
Complete remission												
Yes	595 (91)	180 (91)	48 (98)	7 (78)	42 (81)	29 (83)	20 (95)	14 (100)	13 (100)	11 (92)	6 (100)	139 (94)
No	57 (9)	17 (9)	1 (2)	2 (22)	10 (19)	6 (17)	1 (5)	0 (0)	0 (0)	1 (8)	0 (0)	9 (6)
Outcome at 3 years (95% CI)												
Relapse rate	32% (28–37)	31% (24–39)	50% (36–66)	50% (20–89)	53% (37–71)	56% (38–76)	60% (36–85)	38% (18–69)	26% (9–61)	0%^4^	0%	25% (18–34)
Event free	47% (43–51)	46% (39–53)	38% (25–52)	33% (8–62)	20% (10–33)	28% (15–44)	19% (6–38)	49% (22–72)	54% (25–76)	100%^4^	67% (19–90)	55% (47–63)
Overall survival	54% (50–58)	57% (50–64)	46% (31–59)	44% (14–72)	22% (11–34)	36% (20–52)	24% (9–43)	54% (25–77)	54% (25–76)	100%^4^	67% (19–90)	63% (54–70)
Non-relapse mortality	29% (25–33)	32% (25–39)	20% (11–37)	33% (12–72)	53% (36–72)	34% (20–53)	46% (25–73)	21% (5–63)	27% (9–63)	0%	33% (10–81)	25% (18–33)
Hazard Ratio (95% CI), *p* value^10^												
Relapse rate	–	0.89 (0.65–1.22), 0.473	1.67 (1.10–2.54), 0.015	1.18 (0.38–3.69), 0.779	1.78 (1.11–2.87), 0.018	1.70 (1.00–2.89), 0.050	2.39 (1.30–4.42), 0.005	1.30 (0.57–2.93), 0.531	0.61 (0.20–1.92), 0.402	0.32 (0.08–1.28), 0.106	0.46 (0.06–3.25), 0.433	0.58 (0.40–0.83), 0.003
Event-free	–	1.04 (0.84–1.31), 0.700	1.18 (0.84–1.66), 0.347	1.26 (0.56–2.83), 0.570	1.86 (1.34–2.60), <0.001	1.48 (0.99–2.20), 0.057	2.00 (1.26–3.18), 0.003	0.90 (0.45–1.81), 0.766	0.66 (0.30–1.49), 0.321	0.18 (0.04–0.72), 0.015	0.74 (0.24–2.31), 0.608	0.66 (0.51–0.86), 0.002
Overall	–	0.89 (0.69–1.13), 0.332	1.21 (0.85–1.73), 0.294	1.42 (0.63–3.20), 0.392	2.15 (1.53–3.02), <0.001	1.54 (1.01–2.34), 0.043	2.11 (1.29–3.46), 0.003	0.74 (0.33–1.65), 0.459	0.81 (0.36–1.83), 0.619	0.22 (0.05–0.88), 0.033	0.61 (0.15–2.47), 0.492	0.71 (0.54–0.93), 0.013

Notes: (1) Patients with failed or unknown cytogenetics not included; (2) Includes two patients with near-haploid karyotypes (<30 chromosomes); (3) JAK-STAT abnormalities comprise *IGH-CRLF2* (*n* = 24), *P2RY8-CRLF2* (*n* = 9) and *JAK2* fusions (*n* = 2, *PAX5-JAK2* & *BCR-JAK2*). *P2RY8-CRLF2* fusions were identified by MLPA (*n* = 4), FISH (*n* = 3) or both techniques (*n* = 2); (4) *ZNF384* partner genes included *EP300* (*n* = 5), *TCF3* (*n* = 4), *AKAP8* (*n* = 1), *EWSR1* (*n* = 1), unknown (*n* = 1). No patient had an event before 3 years, 2 patients relapsed one at 3.11 years and one at 6.33 years and both have died; (5) ABL-class fusions comprised *EBF1-PDGFRB* (*n* = 2), *FOXP1-ABL1* (*n* = 1), *PDGFRB/CSF1R* rearrangement (*n* = 2), *ABL1*rearrangement (*n* = 1). In the latter 3 cases we were able to exclude the following partner genes: *EBF1*, *ATF7IP*, *SSBP2* and *ETV6*; (6) The B-other group includes iAMP21 (*n* = 4) and *ETV6-RUNX1* (*n* = 1); (7) Frequencies were calculated using the actual number of cases tested for each abnormality which varied due to availability of material (see Supplementary Fig. 1); (8) MRD, Minimal residual disease was measured at the end of both induction phases. The status at the end of phase 2 was used to assign risk status. MRD was not performed on all cases at either time-point; (9) allo-SCT, allogeneic stem cell transplant; “other” includes patients who died before post-induction could be delivered or who received off-trial therapy; (10) hazard ratio represents the risk of an event for patients with BCP-ALL in that subgroup compared with all other BCP-ALL cases (except those cases with failed or unknown genetics).

A small majority of the 52 patients with ALL classified as HoTr (29/52, 56%) presented with direct evidence of a clone with <40 chromosomes. Analysis of the remaining 23 cases with a near-triploid clone [cytogenetics (*n* = 23), SNP array (*n* = 14)] revealed features consistent with the HoTr subgroup; i.e., tetrasomy of chromosome 1, 6, 11 and 19 and widespread whole chromosome copy-number neutral loss of heterozygosity (CNN-LOH) [30, 32]. Three cases with a near-triploid clone and CNN-LOH had a modal chromosome number below the standard threshold of 60 chromosomes; the presence of a *TP53* mutation in all three cases confirmed their HoTr status [30]. There was no difference in the demographics or clinical features of patients with HoTr according to type of clone(s) present at diagnosis (Table S3).

The two major genetic subgroups underlying the BCR-ABL1-like/Philadelphia-like gene expression profile are defined by JAK-STAT abnormalities and ABL-class fusions. FISH and MLPA screening of >500 cases revealed a frequency of 6% and 1% respectively (Table 1, Fig. 1, Fig. S1). The JAK-STAT group comprised *IGH-CRLF2* (*n* = 23), *P2RY8-CRLF2* (*n* = 9) and *JAK2* fusions (*n* = 2, *PAX5-JAK2* & *BCR-JAK2*). *P2RY8-CRLF2* fusions were determined by MLPA (*n* = 4), FISH (*n* = 3) or both (*n* = 2). We were able to identify the partner gene in 3/6 cases: *EBF1-PDGFRB* (*n* = 2), *FOXP1-ABL1* (*n* = 1). The remaining three cases had a *PDGFRB or /CSF1R* *fusion* (*n* = 2) or *ABL1* (*n* = 1) rearrangement. A lack of material prevented the identification of the partner gene.

A total of 14 (3%) patients had ALL with *TCF3-PBX1* detected by karyotyping (*n* = 10) and/or FISH analysis (*n* = 10). Karyotyping, FISH and SNP array analysis revealed the unbalanced form of the translocation [der(19)t(1;19)] in 8/14 (57%) cases. Patients with der(19)t(1;19) harbour two normal copies of chromosome 1 and thus have trisomy of chromosome arm 1q (Fig. S2c). SNP array analysis of these 14 patient specimens plus an additional 9 t(1;19) cases from UKALLXII [2] showed that all patients with der(19)t(1;19) harboured heterodisomy of chromosome 1 rather than uniparental disomy of chromosome 1 (Fig. S2a, b); suggesting the translocation arose during the G2 phase of the cell cycle rather following after duplication of a normal copy of chromosome 1 [33]. Comparing the demographics and clinical features of patients with the balanced and unbalanced form of the t(1;19) revealed no significant differences.

Twelve patient samples had a *ZNF384-r* with *EP300* (*n* = 5) and *TCF3* (*n* = 4) being the most prevalent partners along with single cases of *AKAP8-ZNF384* and *EWSR1-ZNF384* and one where the partner gene is unknown.

Chromosomal abnormalities associated with childhood ALL were rare: iAMP21 (*n* = 4), *ETV6-RUNX1* (*n* = 1), and patients were younger (25, 26, 26, 30 and 48 years). There were few strong correlations between genetic abnormalities and demographic features but HeH and *ZNF384-r* were more common in patients ≤40 years old whereas HoTr was more prevalent among patients >40 years (Table 1). In addition, patients with HeH or HoTr had lower WCC, but patients with *KMT2A-r* had elevated WCC.

### Frequency of copy number alterations and correlation with primary chromosomal abnormalities

CNA affecting *IKZF1, CDKN2A/B, PAX5, BTG1, ETV6, EBF1, RB1* and the PAR1 region were determined by MLPA in a representative cohort of 437 patients (Table S2). One or more CNA was observed in 270 (62%) patients. Deletions of *IKZF1* or *CDKN2A/B* occurred in 39% and 37% cases (Table 2, Fig. 1B). *PAX5* alterations and *BTG1* deletions were present in 21% and 11% cases while other deletions were present in <10% cases. A total of 189 *IKZF1* deletions were detected in 170 patients. MLPA detects the full spectrum of deletions ranging from canonical intragenic ex4-7 deletions to whole gene deletions as well as rare deletions (Fig. S3). Among 162 patients with a *CDKN2A/B* deletion, 95 (59%) were monoallelic and 67 (41%) biallelic. The distribution of deletions was not random and pairwise testing revealed numerous genes were co-deleted more often than expected (Fig. 1B). *IKZF1* deletions frequently co-existed with *CDKN2A/B* deletions (*p* < 0.001). *CDKN2A/B* and *PAX5* which are co-located on chromosome 9p were also frequently co-deleted (*p* < 0.001). Ninety-three patients (57% of those with an IKZF1 deletion) fulfilled the criteria for IKZF1plus [*IKZF1* deletion plus *PAX5*, *CDKN2A/B* or PAR1 deletion] [34]. There was no association between the type of *CDKN2A/B* deletion and deletion of the other genes investigated (Fig. S4b).

**Table 2:** Demographic characteristics, clinical features and outcome of 436 UKALL14 patients with B-cell precursor ALL according to the presence of selected copy number alterations.

	Total	*IKZF1* Deletions	*CDKN2A/B* Deletions	*PAX5* alterations^1^	*BTG1* Deletions	*ETV6* Deletions	*RB1* Deletions	*EBF1* Deletions	PAR1 Deletions/ *P2RY8-CRLF2*
Total, *n*(%)									
Sex									
Male	240 (55)	96 (56)	83 (51)	53 (57)	25 (54)	12 (38)	17 (59)	10 (67)	5 (83)
Female	196 (45)	74 (44)	79 (49)	40 (43)	21 (46)	20 (63)	12 (41)	5 (33)	1 (17)
Age									
<40 years	158 (36)	55 (32)	51 (31)	29 (31)	15 (33)	8 (25)	9 (31)	6 (40)	0 (0)
≥40 years	278 (64)	115 (68)	111 (69)	64 (69)	31 (67)	24 (75)	20 (69)	9 (60)	6 (100)
White cell count (x10^9^/L)									
<30 × 10^9^/L	306 (70)	112 (66)	103 (64)	64 (69)	28 (61)	24 (75)	23 (79)	8 (53)	5 (83)
≥30 × 10^9^/L	130 (30)	58 (34)	59 (36)	29 (31)	18 (39)	8 (25)	6 (21)	7 (47)	1 (17)
MRD status post-phase 1^2^									
Positive	187 (61)	86 (66)	66 (54)	35 (52)	18 (60)	15 (60)	7 (33)	5 (45)	4 (80)
Negative	119 (39)	44 (34)	56 (46)	32 (48)	12 (40)	10 (40)	14 (67)	6 (55)	1 (20)
MRD status post-phase 2^2^									
Positive	101 (38)	47 (45)	32 (30)	20 (35)	10 (31)	5 (20)	6 (33)	6 (55)	1 (25)
Negative	168 (62)	58 (55)	73 (70)	37 (65)	22 (69)	20 (80)	12 (67)	5 (45)	3 (75)
UKALL14 trial risk group									
Standard	64 (15)	15 (9)	19 (12)	8 (9)	5 (11)	4 (13)	4 (14)	2 (13)	0 (0)
High	372 (85)	155 (91)	143 (88)	85 (91)	41 (89)	28 (88)	25 (86)	13 (87)	6 (100)
Post-induction treatment^3^									
Chemotherapy	92 (21)	26 (15)	34 (21)	16 (17)	10 (22)	8 (25)	6 (21)	3 (20)	1 (17)
Myeloablative allo-SCT	78 (18)	28 (16)	21 (13)	17 (18)	8 (17)	2 (6)	4 (14)	4 (27)	0 (0)
Reduced intensity allo-SCT	146 (33)	54 (32)	58 (36)	36 (39)	16 (35)	14 (44)	12 (41)	4 (27)	3 (50)
Other	120 (28)	62 (36)	49 (30)	24 (26)	12 (26)	8 (25)	7 (24)	4 (27)	2 (33)
Revised UKALL genetic subtype^4^									
Standard Risk	140 (36)	35 (22)	54 (36)	27 (30)	8 (20)	10 (37)	6 (24)	1 (7)	0 (0)
High risk	46 (12)	3 (2)	17 (11)	3 (3)	1 (2)	1 (4)	1 (4)	0 (0)	0 (0)
Very high Risk	43 (11)	23 (14)	24 (16)	16 (18)	11 (27)	7 (26)	6 (24)	5 (36)	6 (100)
Tyrosine Kinase Sensitive	160 (41)	100 (62)	56 (37)	43 (48)	21 (51)	9 (33)	12 (48)	8 (57)	0 (0)
Complete remission									
Yes	401 (92)	148 (87)	149 (92)	87 (94)	42 (91)	31 (97)	26 (90)	13 (87)	5 (83)
No	35 (8)	22 (13)	13 (8)	6 (6)	4 (9)	1 (3)	3 (10)	2 (13)	1 (17)
Outcome at 3 years (95% CI)									
Relapse Rate	34% (29–39)	35% (27–44)	38% (30–48)	36% (25–48)	40% (26–58)	34% (20–55)	29% (13–58)	27% (9–62)	70% (28–99)
Event free	46% (41–51)	45% (37–52)	42% (34–49)	42% (31–51)	41% (27–55)	50% (32–66)	45% (26–63)	53% (26–74)	17% (1–52)
Overall survival	54% (49–59)	56% (48–63)	49% (41–56)	48% (37–58)	54% (39–67)	53% (34–68)	62% (42–77)	53% (26–74)	17% (1–52)
Nonrelapse mortality	29% (25–34)	30% (24–38)	31% (24–39)	34% (24–45)	29% (18–47)	21% (10–42)	36% (21–56)	27% (11–56)	44% (12–93)
Hazard ratio (95% CI), *p* value^5^									
Relapse Rate	–	1.16 (0.84–1.61), 0.373	1.11 (0.79–1.57), 0.539	1.04 (0.69–1.58), 0.840	1.27 (0.76–2.11), 0.355	1.09 (0.61–1.98), 0.767	0.53 (0.22–1.29), 0.161	1.31 (0.58–2.97), 0.518	2.39 (0.76–7.51), 0.137
Event-free	–	1.16 (0.92–1.47), 0.217	1.14 (0.89–1.47), 0.290	1.17 (0.88–1.57), 0.282	1.14 (0.77–1.67), 0.515	0.88 (0.55–1.42), 0.602	0.87 (0.52–1.47), 0.603	1.18 (0.63–2.22), 0.611	1.92 (0.79–4.67), 0.148
Overall	–	0.94 (0.72–1.21), 0.616	1.16 (0.89–1.52), 0.278	1.21 (0.89–1.66), 0.230	1.01 (0.66–1.55), 0.946	0.92 (0.55–1.53), 0.744	0.90 (0.52–1.58), 0.718	1.23 (0.63–2.40), 0.541	2.25 (0.93–5.48), 0.073

Notes: (1) *PAX5* alterations included one patients with an intragenic amplification; (2) MRD, Minimal residual disease was measured at the end of both induction phases. The status at the end of phase 2 was used to assign risk status. MRD was not performed on all cases at either time-point; (3) allo-SCT, allogeneic stem cell transplant; “other” includes patients who died before post-induction could be delivered or who received off-trial therapy; (4) Genetic subtype: Standard/Low risk patients with those with other genetic abnormalities not classified into one of the other categories. High-risk—patients with any *KMT2A* rearrangement; Very high risk—low hypodiploidy/near-triploidy, complex karyotype or JAK-STAT abnormalities; Tyrosine kinase senstivie—*BCR-ABL1* fusion and ABL-class fusions. (5) hazard ratio represents the risk of an event for patients with that specific copy number alteration compared with all other tested cases.

**Fig. 2:**
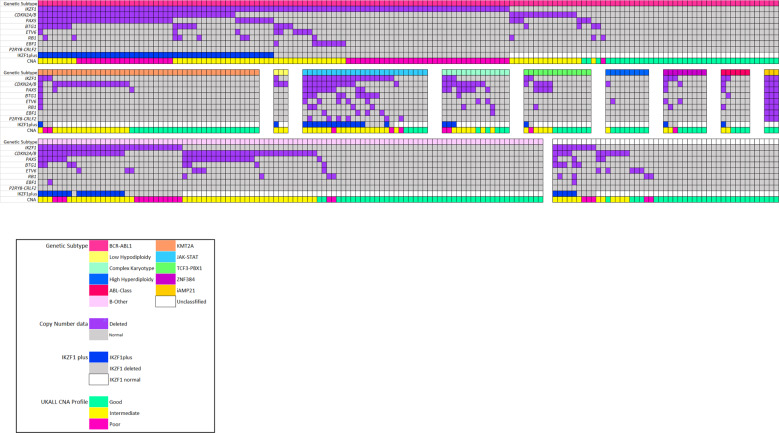
Relationship between selected copy number alterations and genetic subtype in adults with B-cell precursor ALL enroled to UKALL14. Oncoplot showing the pattern of individual copy number alterations, IKZF1plus profile and the UKALL-CNA profile per case according genetic subtype. Purple cells indicate the presence of a deletion affecting that gene. Notes: (1) Patients with low hypodiploidy were under-represented in the tested cohort due to a high failure rate. Calling copy number alterations by MLPA on a backdrop of large-scale chromosomal loss and ploidy doubling is a recognised limitation of the assay (Genes, Chromosomes & Cancer 2010, 49:1104); (2) *PAX5* alterations included a single case with an intragenic *PAX5* amplification (Blood Advances 2017, 1:1473); (3) Among 170 cases with an *IKZF1* deletion, 93 were classified as fulfilling the definition of IKZF1plus (*IKZF1* deletion with concomitant *CDKN2A/B*, *PAX5* or PAR1 deletion in the absence of an *ERG* deletion). All 93 cases had *IKZF1* deletion with concomitant *CDKN2A/B*, *PAX5* or PAR1 deletion. Among these cases, 68 cases with an established primary genetic abnormality were assumed to lack an *ERG* deletion as they are extremely rare in the absence of *IGH-DUX4*. A further 18 cases tested negative for an *ERG* deletion by MLPA. The remaining 7 cases, who were not tested due to lack of material, were included on the basis that *ERG*deletions are rare in B-other (4/150, <3%) and hence <1 case is likely be incorrectly classified.

**Fig. 3:**
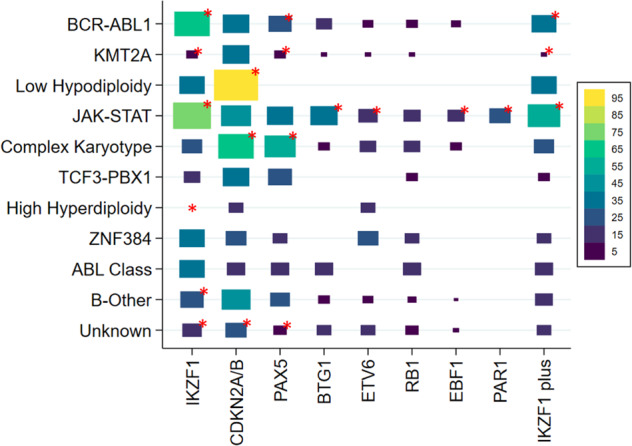
Box plot showing the frequency of copy number alterations and the IKZF1plus profile in selected genetic subtypes. The size and colour of each box represents the proportion of cases in each genetic subtype that have the copy number alteration in question (see legend). The red asterisks marks the significant associations (*p* < 0.05). PAR1 = PAR1 deletions aka *P2RY8-CRLF2*.

There was a strong correlation between primary genetic subtype and the spectrum of CNA (Figs. 2 and 3). Patients with *BCR-ABL1* had a higher frequency of *IKZF1* deletions and IKZF1plus compared to other cases: 60% v 22%, *p* < 0.01 and 32% v 16%, *p* < 0.01. Patients with *KMT2A-r* had few recurrent deletions and 26 (56%) harboured none of these deletions. The only CNA present in >3 *KMT2A-r* cases was *CDKN2A/B* deletions in 17/46 (37%) cases. Patients with a JAK-STAT abnormality had a higher frequency of all CNA (Fig. 3); especially *IKZF1* deletions and IKZF1plus 63% v 18%, *p* < 0.01 and 54% v 12%, *p* < 0.01 respectively. Patients with a CK harboured a high frequency of *CDKN2A/B* and/or *PAX5* deletions (10/14, 71%) but few *IKZF1* deletions (3/14). In order to investigate the spectrum of CNAs associated with CK in more detail, we performed SNP array analysis on a cohort of CK cases from UKALL14 (*n* = 9) and UKALLXII (*n* = 10). This genome-wide analysis confirmed the high frequency of *CDKN2A/B* deletions and low frequency of *IKZF1* deletions (Fig. S5a). There was no consistent pattern or profile of CNAs underpinning CK. The most frequently deleted regions were 9p and 6q targeting *CDKN2A/B*, *MTAP, BACH2* and *EPHA7* (Fig. S5c). There were few correlations between CNA and demographic or clinical features (Table 2). The exception was a female predominance among patients with an *ETV6* deletion (21 females: 12 males, *p* = 0.018).

### Outcome of patients by genetic subtype and prognostic impact of secondary abnormalities

After a median follow-up time of 4.45 years, the 3-year survival rates for the whole BCP-ALL cohort were OS 54% (95% CI 50–58%), EFS 47% (43–51%), RR 32% (28–37%) (Table 1). In univariable analysis, patients with HoTr or CK had double the rate of relapse and death compared with other patients. The OS rates were 22% (11–34%) and 24% (9–43%) respectively despite being treated as high risk and in the case of HoTr having a relatively good response to induction therapy (Table 1). Patients with *KMT2A-AFF1* had an increased RR but not a worse OS. There was no difference in outcome for patients with HoTr according to the ploidy of the presenting clone. However, there was some suggestion that patients presenting with low hypodiploid clones had lower CR rates and higher non-relapse mortality (Table S3).

Patients with *BCR-ABL1* did not have a higher RR or poorer OS compared to the remaining patients (hazard ratio 0.89 (95% CI 0.65–1.22), *p* = 0.5 and 0.89 (0.69–1.13), *p* = 0.3, respectively). Among patients with abnormalities underpinning BCR-ABL1-like, the 6 patients with an ABL-class fusion did not have a poor outcome—all achieved a complete remission post phase 2 and none relapsed within three years of diagnosis. The ABL-class fusion was identified retrospectively so did not influence therapy. MRD was evaluated for four patients and three were negative. In contrast, the 35 patients with a JAK-STAT abnormality had a high rate of MRD positivity at both time-points (88% & 76%), high RR (56%) and a poor OS (36%) despite 83% being treated as high-risk (Table 1).

Patients with *TCF3-PBX1* had outcome almost identical to the cohort overall with RR and OS rates of 38% and 54% respectively. The RR among patients with the balanced and unbalanced form of the translocation was very similar (40% v 38%). Although the *ZNF384* subgroup was small (*n* = 12) none suffered an event within 3 years of diagnosis. Two patients relapsed after 3 years, one at 3.1 years and one at 6.3 years and both died within a year of relapse.

Overall none of the CNA detected by MLPA were associated with survival (Table 2). As previously reported, *IKZF1* deletions did not confer an inferior outcome in *BCR-ABL1* positive, *BCR-ABL1* negative or B-other ALL (Table S4) [35]. Extending our analysis to examine the type of deletion (exons 4–7 and whole gene) and IKZF1plus, again did not reveal any association with outcome (Table S4). Patients harbouring *P2RY8-CRLF2* had a high RR consistent with the outcome of the broader JAK-STAT subgroup (Table 1). We found no evidence of a prognostic effect associated with the UKALL-CNA profile [36, 37] in the whole cohort or the B-other subgroup (Table S4). However the presence of a biallelic *CDKN2A/B* deletion was associated with a poor outcome in the *BCR-ABL1* cohort but not the remaining cases (Table S4, Fig. S4c, d). A comparison of the outcome of patients with biallelic and monoallelic deletions and *BCR-ABL1* revealed raised, but not significant, hazard ratios (Table S4). However, the presence of a biallelic *CDKN2A/B* deletion was associated with lower EFS and OS rates when compared with patients with no *CDKN2A/B* deletion but not higher RR (EFS hazard ratio 1.93 (1.14–3.24), *p* = 0.01 and OS 1.88 (1.06–3.35), *p* = 0.03). A multivariate EFS model with variables for *CDKN2A\B* and *BCR-ABL1* status revealed significant interaction indicating that the prognostic effect of biallelic *CDKN2A/B* deletion was different in *BCR-ABL1* positive and negative cases (*p* = 0.046).

### Revised genetic risk classification for adult ALL

**Fig. 4:**
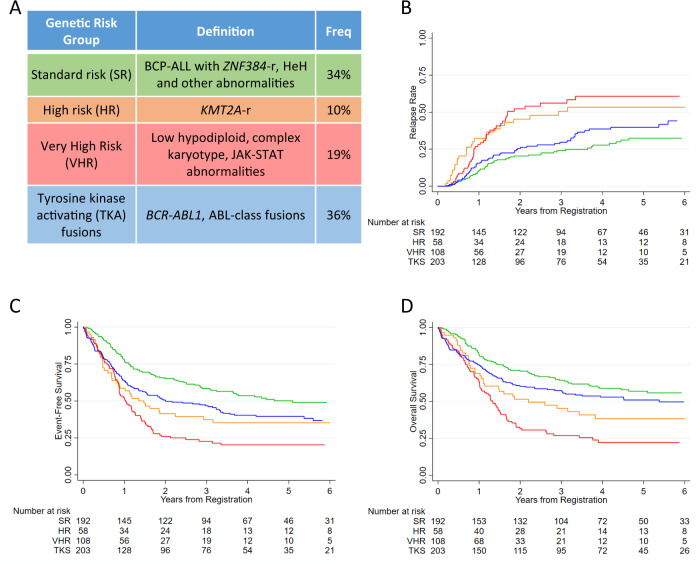
A revised genetic risk classification system for adult ALL. **A** Definition and frequency of the five genetic risk groups; (**B**–**D**) Kaplan–Meier survival curves showing the risk of relapse, event-free survival and overall survival of patients treated in UKALL14 according to this revised genetic classification.

We propose a revised genetic classification for adult ALL comprising five subgroups with distinct genetics and/or outcomes (Table 3, Fig. 4). The very high-risk (VHR) category includes patients with HoTr and CK who had an OS rates <25% at 3 years despite being classified and treated as high risk. Patients with JAK-STAT abnormalities are also included in the VHR group on the basis of a high RR and MRD rates. Stratifying the VHR group according to MRD status post phase 2 induction revealed a high RR at 3 years for both MRD negative and MRD positive cases: 54% (35–76) and 81% (59–95) hazard ratio 1.79 (0.85–3.79), *p* = 0.126. The high-risk group comprises all patients with a *KMT2A* fusion. Although only *KMT2A-AFF1* was classified as high risk on UKALL14, 50% of patients with other *KMT2A* fusion partners relapsed. *KMT2A-r* were not grouped in the VHR group because their OS rates were >40%. Patients in the HR group (i.e. *KMT2A-r*) had a differential RR at 3 years according to MRD status: MRD positive 69% (46-89) v MRD negative 9% (1–49); hazard ratio 6.80 (95% CI 1.49-30.92), *p* = 0.013. Patients with *BCR-ABL1* and ABL-class fusion were combined on the basis of the underlying functional consequence of the fusions and likely sensitivity to a tyrosine kinase inhibitor. The remaining cases which encompass B-other ALL and abnormalities associated with paediatric ALL/younger age are categorised together in standard risk. The majority of patients in the new standard risk group were MRD negative post-induction (74/103, 72%). Among patients in the standard risk group there was no significant difference in RR at 3 years between patients who were MRD positive and negative: 34% (18–57) v 21% (13–32), hazard ratio 1.56 (0.72–3.40), *p* = 0.263. In comparison with the standard genetic risk group, patients in the high-risk genetic group had inferior outcomes in univariable but not in multivariable analysis (Table 3). Whereas the inferior outcome of patients with very high-risk genetics was independent of sex, age and WCC. Patients with tyrosine kinase activating fusions had significantly decreased EFS but not increased RR in relation to standard risk patients. The small number of patients in each revised genetic group receiving protocol defined post-induction therapy makes estimating the interaction between genetics and treatment difficult especially as several genetic abnormalities were used to assign patients to high-risk therapy (Table 3). The OS of patients in the new standard risk genetic group was 64% (57–71) at 3 years significantly higher than other patients 47% (41–52), hazard ratio 1.65 (1.27–2.12), *p* < 0.001. One third of these patients (*n* = 62) received chemotherapy maintenance per protocol and achieved a 3 years OS rate of 72% (57–82).

**Table 3:** Demographic characteristics, clinical features and outcome of UKALL14 patients according to the revised UKALL genetic risk groups.

	Genetic risk group^1^			
	Standard risk	High risk	Very high risk	Tyrosine kinase activating fusions
Total, *n* (%)	192 (34)	58 (10)	108 (19)	203 (36)
Sex				
Male	112 (58)	26 (45)	58 (54)	113 (56)
Female	80 (42)	32 (55)	50 (46)	90 (44)
Age				
<40 years	79 (41)	19 (33)	27 (25)	60 (30)
≥40 years	113 (59)	39 (67)	81 (75)	143 (70)
White cell count (×10^9^/L)				
<30 × 10^9^/L	167 (87)	11 (19)	94 (87)	129 (64)
≥30 × 10^9^/L	25 (13)	47 (81)	14 (13)	74 (36)
MRD status post-phase 1^2^				
Positive	54 (48)	25 (71)	46 (75)	108 (67)
Negative	59 (52)	10 (29)	15 (25)	53 (33)
MRD status post-phase 2^2^				
Positive	29 (28)	16 (57)	28 (52)	55 (39)
Negative	74 (72)	12 (43)	26 (48)	87 (61)
UKALL14 trial risk group				
Standard	56 (29)	0 (0)	6 (6)	2 (1)
High	136 (71)	58 (100)	102 (94)	201 (99)
Post-induction treatment^3^				
Chemotherapy	62 (32)	6 (10)	17 (16)	18 (9)
Myeloablative allo-SCT	23 (12)	10 (17)	14 (13)	48 (24)
Reduced intensity allo-SCT	69 (36)	19 (33)	38 (35)	71 (35)
Other	38 (20)	23 (40)	39 (36)	66 (33)
Complete remission				
Yes	182 (95)	55 (95)	91 (84)	186 (92)
No	10 (5)	3 (5)	17 (16)	17 (8)
Outcome at 3 years (95% CI)				
Relapse rate	24% (18–31)	51% (37–66)	56% (45–68)	30% (23–38)
Event free	58% (51–65)	37% (25–50)	23% (15–31)	47% (40–54)
Overall survival	64% (57–71)	45% (32–58)	27% (19–36)	57% (50–64)
Non-relapse mortality	22% (17–29)	22% (12–36)	44% (34–56)	32% (26–39)
Hazard ratio (95% CI), *p* value^4^				
Relapse rate	1	2.33 (1.45–3.74), <0.001	2.67 (1.78–4.00), <0.001	1.34 (0.92–1.96), 0.130
Event-free	1	1.68 (1.82–3.29), 0.008	2.45 (1.82–3.29), <0.001	1.48 (1.13–1.93), 0.005^5^
Overall	1	1.66 (1.11–2.48), 0.013	2.57 (1.88–3.50), <0.001	1.27 (0.95–1.71), 0.107
Adjusted hazard ratio (95% CI), *p* value^6^				
Relapse rate	1	1.49 (0.81–2.76), 0.210	2.61 (1.74–3.92), <0.001	1.22 (0.83–1.80), 0.304
Event-free	1	1.22 (0.76–1.97), 0.409	2.34 (1.74–3.15), <0.001	1.35 (1.03–1.78), 0.03
Overall	1	1.27 (0.77–2.10), 0.355	2.43 (1.78–3.31), <0.001	1.15 (0.85–1.55), 0.366

Notes (1) Genetic subtype: Standard/Low risk patients with those with other genetic abnormalities not classified into one of the other categories. High-risk - patients with any *KMT2A* rearrangement; Very high risk—low hypodiploidy/near-triploidy, complex karyotype or JAK-STAT abnormalities; Tyrosine kinase sensitive—*BCR-ABL1* fusion and ABL-class fusions. Patients with failed or missing cytogenetics have been excluded (*n* = 122). (2) MRD, Minimal residual disease was measured at the end of both induction phases. The status at the end of phase 2 was used to assign risk status. MRD was not performed on all cases at either time-point; (3) allo-SCT, allogeneic stem cell transplant; “other” includes patients who died before post-induction could be delivered or who received off-trial therapy; (4) Hazard ratio representing the risk of an event for patients in the high, very high, or tyrosine kinase activating group compared with those patients in the standard risk group; (5) hazard ratio reduces and is no longer significant (1.32 (0.99–1.75), *p* = 0.06) if analysis is restricted to cases recruited after the April 2012 amendment which removed induction asparaginase and halved induction dose of daunorubicin for *BCR-ABL1* positive cases; (6) Hazard ratio adjusted for sex, age and white cell count.

## Discussion

The central aims of this project were (1) to assess the prognostic impact of key genetic abnormalities in a contemporary MRD driven trial (UKALL14) and (2) to revise the UKALL genetic risk classification system for adult ALL. We have assessed the prognostic impact of a wide range of genetic abnormalities and propose a revised genetic classification of adult ALL BCP-ALL (Fig. 4). The four risk groups can be used to evaluate the efficacy of treatment interventions including transplant as well as providing a basis for designing novel risk stratification algorithms. Evaluating this revised classification system in additional datasets will help determine its relevance in the context of other therapies; especially immunotherapies.

Patients in UKALL14 with HoTr, *KMT2A-AFF1* or CK were assigned high risk therapy irrespective of other clinical features or MRD. Despite this intervention, the survival of patients with HoTr or CK remained below 25% at 3 years. The poor outcome associated with HoTr has been reported by both paediatric and adult ALL study groups and given the high frequency of *TP53* mutations in HoTr is perhaps not surprising [26, 32, 38–40]. HoTr can cause diagnostic dilemmas because patients present with one or two of the related clones and when only a near-triploid clone is detected there can be confusion with HeH [30]. We recently published guidance and an algorithm to assist in the diagnosis of HoTr in these scenarios [30].

The association of CK with poor outcome has not been observed universally.(37, 41) This outcome heterogeneity is likely due to the subjective nature of the definition of CK. We screened CK cases for known gene fusions but found only one. SNP array analysis of CK cases did not find a consistent or novel profile of CNA underpinning this subgroup. The poor outcome cannot be attributed to *IKZF1* deletions or IKZF1plus which are rare in this subgroup (Fig. 2B). The most frequent CNA observed in CK was *CDKN2A/B* deletions but these are common across all BCP-ALL and not associated with outcome unless present as biallelic deletions which were not common in CK. Further genomic studies are urgently needed to unravel the key genetic drivers of this subtype.

The outcome of patients with *KMT2A-AFF1* and other *KMT2A-r* was very similar which was not the case in UKALLXII [2]. Compared with age-matched counterparts from UKALLXII, patients with *KMT2A-AFF1* treated on UKALL14 had similar RR rates (~50%) but a higher OS rates (45% v < 35%) [4]. It is difficult to compare outcomes across different treatment eras but in UKALL14 patients with *KMT2A-AFF1* were recommended for allo-SCT which was not the case in UKALLXII.

Patients on UKALL14 with *BCR-ABL1* were treated with imatinib added and asparaginase omitted and had an outcome very similar to the overall cohort (Table 1). Although *IKZF1* deletions and IKZF1plus were prevalent in this subgroup those lesions did not have any prognostic effect. A previous GMALL study also showed no effect of *IKZF1* deletions but did report an adverse effect of *CDKNA2/B* deletions in patients undergoing allo-SCT [12]. We did not observe any prognostic effect of *CDKN2A/B* deletions per se but did notice a trend towards an adverse effect for *BCR-ABL1* patients with biallelic *CDKN2A/B* deletions which was not restricted to patients receiving an allo-SCT. There were several key differences between the two studies. The GMALL study was smaller (*n* = 97) recruited patients as young as 18 years old and comprised three different imatinib schedules including “late” imatinib which we have previously shown to be less effective in the UKALLXII trial [41].

We examined the outcome of patients with the two primary genetic drivers of the BCR-ABL1-like subtype. Patients with JAK-STAT abnormalities had a high RR (56% at 3 years) akin to patients with HoTr or CK. Although these patients were not assigned high risk therapy on the basis of genetics, the majority (>80%) received high risk therapy due to other reasons, notably MRD. JAK-STAT abnormalities account for a significant proportion of the BCR-ABL1-like subtype so our results are consistent with previous reports [22–26]. The frequency of ABL-class fusions in this study was low (1%) but consistent with that reported by a US study (2%)[22]. It is impossible to draw definitive conclusions regarding outcome on such a small subgroup but their outcome did not appear to be poor.

*TCF3-PBX1* has previously been reported to be associated with a poor outcome and some clinical study groups classify and treat patients as high risk [40, 42, 43]. However in UKALL14, these patients had an OS rate identical to the whole BCP-ALL cohort (54%) a result consistent with our UKALLXII study [2]. We also report that our small group of 12 patients with *ZNF384-r* had a relatively good outcome with only 2 relapses/deaths reported to date. This finding is consistent with a larger cohort of patients with *ZNF384-r* treated in Japan who had a 5 year OS rate of 74% [44].

We assessed the frequency and prognostic impact of common CNA in adult ALL. The frequency and distribution of the CNA studied in this report were consistent with our previous UKALLXII study [8] and those from other study groups [9, 11–14]. With the exception of a trend for biallelic deletions of *CDKN2A/B* in *BCR-ABL1* cases, we failed to identify any significant associations between CNA and outcome. This was true for individual deletions and CNA profiles in both the whole cohort and specific subgroups. This finding is in contrast to other studies which reported prognostic associations for several CNA notably *IKZF1* and *CDKN2A/B* deletions [8, 9, 11, 13, 14]. The majority of these studies used MLPA as the primary or validation technique so it is unlikely that diverse methodologies are driving these differences. This is supported by the fact that we did not observe any prognostic effect of *IKZF1* deletions in UKALL14 using PCR [35]. However, there are major differences between UKALL14 and other studies with respect to cohort size, age range of the patients and use of SCT. We examined the prognostic effect of CNA among 436 patients aged 25–65 years including 163 patients who were *BCR-ABL1* positive. Previous studies were significantly smaller (mostly < 300 patients) and included younger patients (15–24 years) [8, 9, 11–14]. As the incidence of ALL decreases sharply between 15 and 24 years of age [45] many of the previous cohorts includes large number of cases not eligible for UKALL14. For example, 40% of patients included in our previous UKALLXII study were aged under 25 years old [8]. This point, coupled with the small size of the previous studies and the low frequency of some CNA deletions, means that previous studies were ill-equipped to assess the prognostic impact of CNA in adults aged >25 years old. Our previous UKALLXII study included 51 patients with an *IKZF1* deletion >25 years old much lower than the 170 patients in this study. In addition, the CNA examined in this study and previous studies are based on deletions common in high-risk children (43) and may not represent the key CNA in adult ALL. Our findings are substantially more robust and pertinent to the wider group of adults with ALL. This is an important observation given that many adolescent and young adult patients are increasingly being treated on paediatric inspired protocols and future adult ALL protocols are likely to have age inclusion criteria more akin to UKALL14.

In conclusion, the extensive analysis of genetic data for adults treated on UKALL14 confirmed the high-risk status of HoTr, *KMT2A-r* and CK while adding JAK-STAT abnormalities to the list of high-risk abnormalities in adult ALL. We have proposed a revised UKALL genetic classification for adult ALL which will enhance future studies.

## Supplementary information

Supplementary Material
