# Supplementary material for: Prognostic impact of chromosomal abnormalities and copy number alterations in adult B-cell precursor acute lymphoblastic leukaemia: a UKALL14 study
Source: Leukemia. 2021 Oct 16;36(3):625–36. doi: 10.1038/s41375-021-01448-2 (PMC8885405; doi:10.1038/s41375-021-01448-2)
Supplement: Supplementary file 1 — Supplementary Material [file 41375_2021_1448_MOESM1_ESM.pdf]

Supplementary methods

Patients were enrolled onto UKALL14 (ISRCTN 66541317), a multi-centre phase 3 clinical trial for patients (25-65 years old) with *de novo* ALL, between December 2010 and July 2018. Five patients aged <25 years with *BCR-ABL1* positive ALL were also recruited. All patients provided written informed consent to trial treatment and correlative science studies. Patients underwent a two-phase induction followed by stratification to continuing chemotherapy or allogeneic stem cell transplant (allo-SCT) based on risk assessment. Patients were assigned to high-risk treatment if they had any of the following features: *BCR-ABL1*/t(9;22)(q34;q11), *KMT2A-AFF1*/t(4;11)(q21;q23), HoTr (30-39 / 60-78 chromosomes), CK (5 or more chromosomal abnormalities), presenting WCC >30x10^9/l, any level of MRD at the end of the second phase of induction and age ≥41 years. High-risk patients were assigned to allo-SCT if they were fit and had an antigen-matched sibling or unrelated donor. Conditioning was myeloablative for patients aged ≤40 years. The safety and efficacy of a reduced intensity conditioned allo-SCT was evaluated as a trial question for patients who were ≥41 years old. Patients with B-ALL were randomized between standard-of-care or standard-of-care plus four doses of rituximab given during the first phase of induction.

Pre-treatment genetic analysis was performed on diagnostic bone marrow samples by regional NHS genetic laboratories. Cytogenetic and florescence in situ hybridisation (FISH) results along with left-over fixed cell suspensions were collected by the Leukaemia Research Cytogenetics Group at Newcastle University.[1] Additional interphase FISH was performed on these fixed cells using commercially available break-apart probes (Table S1). MRD analysis detected and quantified patient specific immunoglobulin (Ig) and T-cell receptor (TCR) gene re-arrangements for *BCR-ABL1* negative patients. The UK Adult ALL MRD lab (UCL Cancer Institute) processed all samples and results were generated and interpreted according to EuroMRD guidelines.[2] MRD was measured after both phases of induction but only the post-phase 2 results were used to assign treatment. Multiplex Ligation-dependent Probe Amplification (MLPA) and SNP array analysis was performed on DNA extracted from pre-treatment bone marrow samples using the SALSA MLPA P335 (ALL-IKZF1), P327 (iAMP21-ERG) kits (MRC Holland, The Netherlands) or the Illumina CytoSNP 850k platform (Illumina, San Diego, CA, USA). All analysis was performed as previously described.[1, 3] Illumina-generated IDAT files were first processed using the GenomeStudio 2.0 (Illumina, San Diego, CA, USA), then loaded into Nexus Copy Number 10 (Biodiscovery, El Segundo, CA, USA) for analysis. Copy number data derived from MLPA analysis was used to classify patients according to the following predefined profiles: UKALL-CNA (good, intermediate and poor groups)[4, 5] and IKZF1plus [6]. To boost the number of samples for SNP array

analysis, we included 19 samples taken from UKALLXII patients who had complex karyotype and *TCF3-PBX1* cases. These cases were not included in cohort used for frequency calculations, clinical associations or outcome analysis.

Event-free survival (EFS) was defined as time from randomisation to relapse, second tumour or death, censoring at date of last contact. Relapse rate (RR) was defined as time from remission to relapse for those achieving a complete remission, censoring at date of death in remission or last contact. Overall survival (OS) was defined as time to death, censoring at date of last contact. All survival rates are quoted at 3 years. Kaplan-Meier methods were used to estimate survival rates and the two-sided log-rank test was employed to evaluate the equality of the survivorship functions in different subgroups. Hazard ratios were estimated using univariable and multivariable Cox regression models. Other comparisons were performed using χ^2 or Fisher's exact test as appropriate. All P values were two-sided and, because of multiple testing, values <0.01 were considered statistically significant. All analyses were performed using Intercooled Stata v165 (StataCorp, College Station, TX) and R version 3.4.3 (http://www.R-project.org).

References

1. Moorman AV, Schwab C, Ensor HM, Russell LJ, Morrison H, Jones L*, et al.* IGH@ translocations, CRLF2 deregulation, and microdeletions in adolescents and adults with acute lymphoblastic leukemia. *J Clin Oncol* 2012 Sep 1; **30**(25)**:** 3100-3108.

2. Bruggemann M, Schrauder A, Raff T, Pfeifer H, Dworzak M, Ottmann OG*, et al.* Standardized MRD quantification in European ALL trials: proceedings of the Second International Symposium on MRD assessment in Kiel, Germany, 18-20 September 2008. *Leukemia* 2010 Mar; **24**(3)**:** 521-535.

3. Creasey T, Enshaei A, Nebral K, Schwab C, Watts K, Cuthbert G*, et al.* Single nucleotide polymorphism array-based signature of low hypodiploidy in acute lymphoblastic leukemia. *Genes, chromosomes & cancer* 2021 Sep; **60**(9)**:** 604-615.

4. Hamadeh L, Enshaei A, Schwab C, Alonso CN, Attarbaschi A, Barbany G*, et al.* Validation of the United Kingdom copy-number alteration classifier in 3239 children with B-cell precursor ALL. *Blood Adv* 2019 Jan 22; **3**(2)**:** 148-157.

5. Moorman AV, Enshaei A, Schwab C, Wade R, Chilton L, Elliott A*, et al.* A novel integrated cytogenetic and genomic classification refines risk stratification in pediatric acute lymphoblastic leukemia. *Blood* 2014 Aug 28; **124**(9)**:** 1434-1444.

6. Stanulla M, Dagdan E, Zaliova M, Moricke A, Palmi C, Cazzaniga G*, et al.* IKZF1(plus) Defines a New Minimal Residual Disease-Dependent Very-Poor Prognostic Profile in Pediatric B-Cell Precursor Acute Lymphoblastic Leukemia. *J Clin Oncol* 2018 Apr 20; **36**(12)**:** 1240-1249.

Supplementary Table 1: Details of commerical and home-grown FISH probes used in this study

Gene	Probe Name	Probe manufacturer*
JAK2	Kreatech™ JAK2 (9p24) Break FISH probe KBI-10012	Kreatech
PDGFRB	PDGFRB, 5q32, Red(3'); PDGFRB, 5q32, Green (5') LPH 031	Cytocell
ABL2	myProbes®Cytocell 2 red probes(186 and 177Kb) and 2 green probes (161 and 199Kb) break apart	Cytocell
ABL1	myProbes® Cytocell break apart	Cytocell
CRLF2	CRLF2, Xp22.33/Yp11.32, Green (3'), CRLF2, Xp22.33/Yp11.32, Red (5') RU-LPH 085	Cytocell
ZNF384	myProbes®Cytocell 12p13.31 ,160Kb (Green 3')/12p13.31, 137 Kb (Red 5')	Cytocell
MEF2D	myProbes® Cytocell 147Kb (red) 129Kb (green)	Cytocell
IGH	IGHC, 14q32.33, Red IGHV, 14q32.33, Green; LPH 014-20	Cytocell
TCF3	E2A, 19p13.3, Red E2A 5', 19p13.3, Green 3' LPH 019	Cytocell
EP300	myProbes® Cytocell break apart	Cytocell
EWSR1	EWSR1, 22q12.1-q12.2, (Red) 5'/ EWSR1, 22q12.1-q12.2, (Green) 3' LPS 006	Cytocell
CREBBP	ZytoLight SPEC CREBBP Dual Color Break Apart Probe (9Z-2267-50)	Zyto
ATF7IP	myProbes® Cytocell break apart	Cytocell
SSBP2	myProbes® Cytocell break apart	Cytocell
BCR	BCR FISH DNA Probe, Split Signal Y5403	Dako
CEBPA	CEBPA/G RP11-475K23 start: 33,379,992; end: 33,528,442; length 148,451 19q13.11 CentromericRed CEBPA/G RP11-270I13 start: 33,674,047 end: 33,855,563 length: 181,517 19q13.11 CentromericRedCEBPA/G RP11-547I3 start: 33,778,074 end: 33,941,111; length: 163,038 19q13.11 TelomericGreenCEBPA/G RP11-423J18 start: 33,958,632 end: 34,156,514 length: 197,883 19q13.11 TelomericGreen	Home-grown
CEBPB	CEBPB RP4-710H13 start: 48,578,865 end: 48,658,543 length: 79,778 20q13.13 CentromericGreen CEBPB RP5-1185N5 start: 48,658,544; end: 48,772,032 length 113,588 20q13.13 CentromericGreenCEBPB RP11-290F20 start: 48,865,332 end: 49,025,542 length: 160,210 20q13.13TelomericRed CEBPB RP5-894K16 start: 49,025,543 end: 49,157,049 length: 131,606 20q13.13 TelomericRed	Home-grown

* Addresses: Kreatech Biotechnology B.V. Vlierweg 20 1032 LG Amsterdam The Netherlands; Cytocell Ltd. 3-4 Technopark Newmarket Road Cambridge, CB5 8PB, UK; Generon Ltd. 11, Progress Business Centre, Whittle Parkway, Slough SL1 6DQ, UK; Agilent Technologies LDA UK Limited 5500 Lakeside Cheadle Royal Business Park Cheadle, Cheshire SK8 3GR

Supplementary Table 2: Demographic characteristics, clinical features and outcome of UKALL14 patients with B-cell precursor ALL according to availablity of material for MLPA tetsing.

	Total BCP-ALL	Tested by MLPA	Not Tested by MLPA	Successful	p-value for tested v not-tested
Total, n(%)	652 (100)	495 (76)	157 (24)	436 (67)	
Sex					
Male	358 (55)	269 (54)	89 (57)	240 (55)	0.624
Female	294 (45)	226 (46)	68 (43)	196 (45)	
Age					
<40	213 (33)	167 (34)	46 (29)	158 (36)	0.302
≥40	439 (67)	328 (66)	111 (71)	278 (64)	
White Cell Count					
<30	484 (74)	353 (71)	131 (83)	306 (70)	0.002
≥30	168 (26)	142 (29)	26 (17)	130 (30)	
UKALL14 Trial Risk Group					
Standard	87 (13)	66 (13)	21 (13)	64 (15)	0.989
High	565 (87)	429 (87)	136 (87)	372 (85)	
Genetic Abnormality					
BCR-ABL1	197 (30)	163 (33)	34 (22)	154 (36)	<0.001
KMT2A rearrangement	58 (9)	48 (10)	10 (6)	46 (11)	
Low hypodiploidy	52 (8)	33 (7)	19 (12)	3 (1)	
JAK-STAT	35 (5)	30 (6)	5 (3)	26 (6)	
Complex Karyotype	21 (3)	15 (3)	6 (4)	14 (3)	
TCF3-PBX1	14 (2)	14 (3)	0 (0)	14 (3)	
High Hyperdiploidy	13 (2)	10 (2)	3 (2)	9 (2)	
ZNF384 rearrangement	12 (2)	9 (2)	3 (2)	9 (2)	
ABL-class fusion	6 (1)	6 (1)	0 (0)	6 (1)	
Remaining cases	244 (37)	167 (34)	77 (49)	155 (35)	
Survival 3yrs, (95% CI)					
Relapse Rate	32% (28-37)	35% (30-40)	25% (18-34)	34% (29-39)	0.062
Event-Free	47% (43-51)	45% (41-50)	53% (44-60)	46% (41-51)	0.083
Overall	54% (50-58)	52% (48-57)	59% (51-66)	54% (49-59)	0.223

Notes: Patients with low hypodiploidy were under-represented in the tested cohort due to a high failure rate. Calling CNA by MLPA on a backdrop of large-scale chromosomal loss and ploidy doubling is a recognised limitation of the assay. (Genes Chromosomes & Cancer. 2010;49(12):1104-1113)

Supplementary Table 3: Demographic characteristics, clinical features and outcome of UKALL14 patients with B-cell precursor ALL belonging to the low hypodiploid / near-triploidy subgroup according to the presenting clone.

	Total	Presented with a …		P value
		Low hypodiploid +/- a near-triploid clone [1]	Near triploid clone only	
Total, n(%)	52 (100)	29 (56)	23 (44)	
Sex				
Male	26 (50)	14 (48)	12 (52)	0.8
Female	26 (50)	15 (52)	11 (48)	
Age				
<40 years	7 (13)	5 (17)	2 (9)	0.4
≥40 years	45 (87)	24 (83)	21 (91)	
White Cell Count ($x10^9/L$)				
$<30x10^9/L$	50 (96)	28 (97)	22 (96)	0.9
$≥30x10^9/L$	2 (4)	1 (3)	1 (4)	
MRD status post-phase 1 [2]				
Positive	16 (64)	6 (50)	10 (77)	0.2
Negative	9 (36)	6 (50)	3 (23)	
MRD status post-phase 2 [2]				
Positive	7 (32)	2 (20)	5 (42)	0.3
Negative	15 (68)	8 (80)	7 (58)	
UKALL14 Trial Risk Group				
Standard	0 (0)	0 (0)	0 (0)	
High	52 (100)	29 (100)	23 (100)	
Post-induction treatment [3]				
Chemotherapy	4 (8)	1 (3)	4 (8)	0.08
Myeloablative allo-SCT	6 (12)	5 (17)	6 (12)	
Reduced intesnity allo-SCT	20 (38)	8 (28)	20 (38)	
Other	22 (42)	15 (52)	22 (42)	
Complete remission				
Yes	42 (82)	22 (79)	20 (87)	0.4
No	9 (18)	6 (21)	3 (13)	
Outcome at 3 years (95% CI)				
Relapse Rate	53% (37-71)	38% (21-62)	71% (47-91)	
Event Free	20% (10-33)	23% (9-41)	17% (5-35)	
Overall survival	22% (11-34)	23% (9-41)	22% (8-40)	
Non-relapse mortality	53% (36-72)	62% (40-84)	38% (21-62)	
Hazard Ratio (95% CI), p value				
Relapse Rate	-	1	1.75 (0.70-4.37)	0.228
Event-Free	-	1	1.18 (0.63-2.20)	0.601
Overall	-	1	1.00 (0.53-1.88)	0.998

Notes (1) Includes two patients with near-haploid karyotypes (<30 chromosomes); (2) MRD, Minimal residual disease was measured at the end of both induction phases. The status at the end of phase 2 was used to assign risk status. MRD was not performed on all cases at either time-point; (3) allo-SCT, allogeneic stem cell transplant; "other" includes patients who died before post-induction could be delivered or who received off-trial therapy.

Supplementary Table 4: Demographic characteristics, clinical features and outcome of UKALL14 patients with B-cell precursor ALL according to the presence of an IKZF1plus profile and by UKALL-CNA profile.

	IKZF1 deletion in *BCR-ABL1* positive cases		*IKZF1* deletion in *BCR-ABL1* negative cases		Type of *IKZF1* deletion			IKZF1plus[1] within cohort of IKZF1 deleted patients		IKZF1plus[1] within whole cohort		IKZF1plus[1] within BCR-ABL1 positive cohort		IKZF1plus[1] within BCR-ABL1 negative cohort		UKALL CNA Profile [2]			UKALL CNA Profile [2] among patients with B-other ALL			Type of *CDKN2A/B* deletion		Biallelic *CDKN2A/B* deletion in *BCR-ABL1* positive cases [7]		Type of CDKN2A/B deletion in BCR-ABL1 negative cases	
	Yes	No	Yes	No	Ex4-7	Ex1-8	Other deletions	Yes	No	Yes	No	Yes	No	Yes	No	Good Risk	Intermediate Risk	High Risk	Good Risk	Intermediate Risk	High Risk	Monoallelic	Biallelic	Normal / Monoallelic	Biallelic	Normal / Monoallelic	Biallelic
Total, n(%)	98 (60)	65 (39)	72 (22)	260 (78)	52 (31)	37 (22)	81 (48)	93 (55)	77 (45)	93 (21)	343 (79)	49 (32)	105 (69)	44 (16)	238 (84)	180 (41)	172 (39)	84 (19)	45 (43)	45 (43)	15 (14)	95 (59)	67 (41)	32 (58)	23 (42)	63 (59)	44 (41)
Sex																											
Male	52 (53)	35 (54)	44 (61)	138 (53)	26 (50)	24 (65)	46 (57)	49 (53)	47 (61)	49 (53)	191 (56)	23 (47)	60 (57)	26 (59)	131 (55)	97 (54)	89 (52)	54 (64)	22 (49)	25 (56)	12 (80)	49 (52)	34 (51)	14 (44)	11 (48)	35 (56)	23 (52)
Female	46 (47)	30 (46)	28 (39)	122 (47)	26 (50)	13 (65)	35 (43)	44 (47)	30 (39)	44 (47)	152 (44)	26 (53)	45 (43)	18 (41)	107 (45)	83 (46)	83 (48)	30 (36)	23 (51)	20 (44)	3 (20)	46 (48)	33 (49)	18 (56)	12 (52)	28 (44)	21 (48)
Age																											
<40 years	24 (24)	18 (28)	31 (43)	94 (36)	15 (29)	11 (30)	29 (36)	23 (25)	32 (42)	23 (25)	135 (39)	8 (16)	33 (31)	15 (34)	102 (43)	72 (40)	55 (32)	31 (37)	14 (31)	17 (38)	8 (53)	35 (37)	16 (24)	9 (28)	2 (9)	37 (59)	30 (68)
≥40 years	74 (76)	47 (72)	41 (57)	166 (64)	37 (71)	26 (70)	52 (64)	70 (75)	45 (58)	70 (75)	208 (61)	41 (84)	72 (69)	29 (66)	136 (57)	108 (60)	117 (68)	53 (63)	31 (69)	28 (62)	7 (47)	60 (63)	51 (76)	23 (72)	21 (91)	26 (41)	14 (32)
White Cell Count (x10^9/L)																											
<30x10^9/L	59 (60)	39 (60)	19 (26)	58 (22)	34 (65)	27 (73)	51 (63)	61 (66)	51 (66)	61 (66)	245 (71)	31 (63)	64 (61)	14 (32)	57 (24)	139 (77)	105 (61)	62 (74)	43 (96)	39 (87)	14 (93)	63 (66)	40 (60)	21 (66)	10 (43)	42 (67)	30 (68)
≥30x10^9/L	39 (40)	26 (40)	53 (74)	202 (78)	18 (35)	10 (27)	30 (37)	32 (34)	26 (34)	32 (34)	98 (29)	18 (37)	41 (39)	30 (68)	181 (76)	41 (23)	67 (39)	22 (26)	2 (4)	6 (13)	1 (7)	32 (34)	27 (40)	11 (34)	13 (57)	21 (33)	14 (32)
MRD status post-phase 1 [3]																											
Positive	51 (65)	39 (72)	35 (67)	83 (54)	29 (71)	17 (57)	40 (68)	37 (54)	49 (79)	37 (54)	150 (63)	17 (49)	68 (76)	20 (61)	82 (55)	65 (60)	75 (58)	47 (69)	14 (61)	16 (46)	6 (67)	41 (57)	25 (50)	13 (57)	10 (56)	28 (57)	15 (47)
Negative	27 (35)	15 (28)	17 (33)	72 (46)	12 (29)	13 (43)	19 (32)	31 (46)	13 (21)	31 (46)	88 (37)	18 (51)	22 (24)	13 (39)	66 (45)	43 (40)	55 (42)	21 (31)	9 (39)	19 (54)	3 (33)	31 (43)	25 (50)	10 (43)	8 (44)	21 (43)	17 (53)
MRD status post-phase 2 [3]																											
Positive	26 (41)	21 (41)	21 (50)	42 (30)	13 (42)	5 (26)	29 (53)	18 (33)	29 (57)	18 (33)	83 (39)	7 (24)	38 (48)	11 (44)	45 (33)	33 (34)	40 (32)	28 (57)	7 (35)	6 (20)	3 (43)	19 (30)	13 (31)	5 (25)	7 (47)	14 (33)	6 (22)
Negative	37 (59)	30 (59)	21 (50)	96 (70)	18 (58)	14 (74)	26 (47)	36 (67)	22 (43)	36 (67)	132 (61)	22 (76)	42 (53)	14 (56)	90 (67)	63 (66)	84 (68)	21 (43)	13 (65)	24 (80)	4 (57)	44 (70)	29 (69)	15 (75)	8 (53)	29 (67)	21 (78)
UKALL14 Trial Risk Group																											
Standard	0 (0)	0 (0)	57 (79)	209 (80)	4 (8)	3 (8)	8 (10)	6 (6)	9 (12)	6 (6)	58 (17)	0 (0)	0 (0)	6 (14)	58 (24)	37 (21)	19 (11)	8 (10)	10 (22)	13 (29)	5 (33)	14 (15)	5 (8)	0 (0)	0 (0)	14 (22)	5 (11)
High	98 (100)	65 (100)	15 (21)	51 (20)	48 (92)	34 (92)	73 (90)	87 (94)	68 (88)	87 (94)	285 (83)	49 (100)	105 (100)	38 (86)	180 (76)	143 (79)	153 (89)	76 (90)	35 (78)	32 (71)	10 (67)	81 (85)	62 (93)	32 (100)	23 (100)	49 (78)	39 (89)
Post-induction treatment [4]																											
Chemotherapy	9 (9)	6 (9)	17 (24)	67 (26)	7 (13)	7 (19)	12 (15)	14 (15)	12 (16)	14 (15)	78 (23)	7 (14)	7 (7)	7 (16)	71 (30)	45 (25)	35 (20)	12 (14)	13 (29)	16 (36)	5 (33)	19 (20)	15 (22)	3 (9)	4 (17)	16 (25)	11 (25)
Myeloablative allo-SCT	18 (18)	19 (29)	10 (14)	39 (15)	8 (15)	5 (14)	15 (19)	12 (13)	16 (21)	12 (13)	66 (19)	5 (10)	31 (30)	7 (16)	35 (15)	37 (21)	24 (14)	17 (20)	4 (9)	4 (9)	1 (7)	15 (16)	6 (9)	6 (19)	2 (9)	9 (14)	4 (9)
Reduced intensity allo-SCT	36 (37)	22 (34)	18 (25)	93 (36)	17 (33)	14 (38)	23 (28)	35 (38)	19 (25)	35 (38)	111 (32)	19 (39)	36 (34)	16 (36)	75 (32)	59 (33)	64 (37)	23 (27)	15 (33)	18 (40)	4 (27)	31 (33)	27 (40)	10 (31)	8 (35)	21 (33)	19 (43)
Other	35 (36)	18 (28)	27 (38)	61 (23)	20 (38)	11 (30)	31 (38)	32 (34)	30 (39)	32 (34)	88 (26)	18 (37)	31 (30)	14 (32)	57 (24)	39 (22)	49 (28)	32 (38)	13 (29)	7 (16)	5 (33)	30 (32)	19 (28)	13 (41)	9 (39)	17 (27)	10 (23)
Revised UKALL Genetic Subtype [5]																											
Standard Risk	0 (0)	0 (0)	35 (56)	113 (52)	10 (21)	8 (24)	17 (22)	19 (22)	16 (22)	19 (22)	121 (40)	0 (0)	0 (0)	19 (49)	121 (62)	70 (47)	53 (33)	17 (22)	45 (100)	45 (100)	15 (100)	30 (33)	24 (39)	0 (0)	0 (0)	30 (52)	24 (63)
High Risk	0 (0)	0 (0)	3 (5)	45 (21)	0 (0)	0 (0)	3 (4)	1 (1)	2 (3)	1 (1)	45 (15)	0 (0)	0 (0)	1 (3)	45 (23)	27 (18)	17 (11)	2 (3)	0 (0)	0 (0)	0 (0)	12 (13)	5 (8)	0 (0)	0 (0)	12 (21)	5 (13)
Very High Risk	0 (0)	0 (0)	23 (37)	55 (25)	6 (13)	1 (3)	16 (20)	18 (20)	5 (7)	18 (20)	25 (8)	0 (0)	0 (0)	18 (46)	25 (13)	10 (7)	28 (18)	5 (6)	0 (0)	0 (0)	0 (0)	15 (17)	9 (15)	0 (0)	0 (0)	15 (26)	9 (24)
Tyrosine Kinase Sensitive	98 (100)	65 (100)	2 (3)	4 (2)	32 (67)	25 (74)	43 (54)	50 (57)	50 (68)	50 (57)	110 (37)	49 (100)	105 (100)	1 (3)	5 (3)	43 (29)	62 (39)	55 (70)	0 (0)	0 (0)	0 (0)	33 (37)	23 (38)	32 (100)	23 (100)	1 (2)	0 (0)
Complete remission																											
Yes	87 (89)	61 (94)	61 (85)	244 (94)	44 (85)	33 (89)	71 (88)	83 (89)	65 (84)	83 (89)	318 (93)	43 (88)	97 (92)	40 (91)	221 (93)	170 (94)	160 (93)	71 (85)	42 (93)	44 (98)	11 (73)	85 (89)	64 (96)	27 (84)	21 (91)	58 (92)	43 (98)
No	11 (11)	4 (6)	11 (15)	16 (6)	8 (15)	4 (11)	10 (12)	10 (11)	12 (16)	10 (11)	25 (7)	6 (12)	8 (8)	4 (9)	17 (7)	10 (6)	12 (7)	13 (15)	3 (7)	1 (2)	4 (27)	10 (11)	3 (4)	5 (16)	2 (9)	5 (8)	1 (2)
Outcome at 3 years (95% CI)																											
Relapse Rate	32% (23-45)	35% (23-51)	37% (26-52)	34% (28-41)	31% (18-49)	35% (20-55)	37% (26-50)	37% (26-50)	32% (21-45)	37% (26-50)	33% (28-39)	33% (20-51)	35% (25-47)	41% (26-61)	32% (26-39)	32% (25-40)	37% (30-46)	31% (21-45)	24% (13-41)	35% (22-54)	23% (8-55)	36% (26-49)	41% (29-56)	35% (19-59)	49% (28-76)	37% (25-52)	37% (23-56)
Event Free	47% (37-57)	43% (31-55)	43% (31-54)	46% (40-52)	45% (31-58)	43% (27-58)	46% (35-56)	39% (29-49)	52% (40-63)	39% (29-49)	48% (43-53)	42% (28-55)	46% (36-56)	36% (22-50)	49% (42-55)	47% (39-54)	44% (37-52)	48% (37-58)	51% (35-64)	45% (30-59)	67% (38-85)	44% (34-54)	38% (27-50)	45% (27-61)	26% (11-45)	43% (31-55)	45% (29-59)
Overall survival	55% (42-66)	59% (48-68)	52% (40-63)	50% (44-56)	58% (43-70)	54% (37-68)	55% (44-66)	48% (37-58)	65% (53-75)	48% (37-58)	56% (50-61)	52% (37-65)	59% (49-68)	43% (28-57)	54% (47-60)	53% (45-60)	53% (45-60)	59% (47-69)	53% (37-66)	54% (39-68)	73% (44-89)	51% (41-61)	45% (33-56)	59% (39-73)	35% (17-54)	48% (35-60)	50% (35-64)
Non-relapse mortality	29% (21-39)	31% (21-44)	30% (20-43)	28% (23-35)	34% (22-48)	33% (19-52)	27% (19-39)	35% (26-46)	23% (15-34)	35% (26-46)	26% (22-32)	33% (22-49)	28% (20-37)	37% (24-54)	26% (21-33)	29% (23-37)	27% (21-35)	29% (21-40)	33% (20-50)	29% (17-46)	13% (4-44)	29% (21-40)	32% (22-46)	29% (16-49)	41% (24-65)	29% (19-43)	28% (16-44)
Hazard Ratio (95% CI), p value [6]																											
Relapse Rate	1.08 (0.61-1.92), 0.789	1	1.23 (0.78-1.92), 0.371	1	1.09 (0.49-2.40), 0.834	1	1.23 (0.60-2.53), 0.568	1.20 (0.71-2.03), 0.505	1	1.28 (0.86-1.91), 0.221	1	0.99 (0.53-1.85), 0.984	1	1.45 (0.85-2.50), 0.176	1	0.88 (0.62-1.27), 0.505	1	0.82 (0.50-1.32), 0.411	0.89 (0.41-1.93), 0.769	1	0.80 (0.26-2.44), 0.698	1	1.23 (0.71-2.12), 0.467	1	1.87 (0.72-4.87), 0.197	1	1.02 (0.51-2.01), 0.965
Event-Free	1.06 (0.71-1.59), 0.780	1	1.21 (0.87-1.68), 0.263	1	1.03 (0.60-1.77), 0.909	1	0.97 (0.59-1.60), 0.910	1.36 (0.92-2.00), 0.122	1	1.40 (1.06-1.87), 0.019	1	1.20 (0.78-1.84), 0.416	1	1.52 (1.03-2.24), 0.037	1	0.97 (0.74-1.27), 0.807	1	1.01 (0.72-1.42), 0.967	1.16 (0.68-1.97), 0.588	1	0.76 (0.33-1.74), 0.513	1	1.20 (0.81-1.78), 0.370	1	1.77 (0.92-3.41), 0.088	1	0.96 (0.58-1.60), 0.890
Overall	0.89 (0.57-1.39), 0.602	1	1.02 (0.71-1.46), 0.914	1	1.02 (0.56-1.86), 0.952	1	0.99 (0.57-1.73), 0.967	1.51 (0.97-2.36), 0.068	1	1.25 (0.92-1.71), 0.156	1	1.16 (0.72-1.87), 0.551	1	1.41 (0.92-2.15), 0.116	1	1.05 (0.78-1.39), 0.765	1	0.89 (0.61-1.31), .564	1.35 (0.77-2.35), 0.298	1	0.74 (0.30-1.81), 0.504	1	1.20 (0.78-1.84), 0.400	1	1.67 (0.81-3.42), 0.163	1	1.00 (0.59-1.70), 0.994

Notes (1) The definition of IKZF1plus is *IKZF1* deletion with concomitant *CDKN2A/B* , *PAX5* or PAR deletion in the absence of an *ERG* deletion. Among 170 cases with an *IKZF1* deletion, 93 had concomitant deletions of *CDKN2A/B* , *PAX5* or PAR. *ERG* deletions are synonymous with *IGH-DUX4* fusion and extremely rare in other genetic subgroups. Among these 93 cases, 68 had an established primary genetic abnormality (*BCR-ABL1* n=49, JAK-STAT n=14, *ZNF384* fusion n=1, *KMT2A* fusion n=1, *TCF3-PBX1* n=1, ABL-class n=1, low hypodiploid n=1) and were assumed not to harbour an ERG deletion. Among the remaining 25 cases, 18 were tested by MLPA (none had an ERG deletion) and 7 were not tested due to lack of material. Thus the final cohort of IKZF1plus cases comprised 93 cases including 7 cases not tested for an *ERG* deletion. (2) UKALL-CNA profile divided cases into three risk groups according to the deletion/alteration status of *IKZF1, CDKN2A/B, PAX5, BTG1, ETV6, EBF1, RB1* and the PAR1 region. Full details can be found in Moorman et al (2014) Blood 124(9):1434-1444. (3) MRD, Minimal residual disease was measured at the end of both induction phases. The status at the end of phase 2 was used to assign risk status. MRD was not performed on all cases at either time-point; (4) allo-SCT, allogeneic stem cell transplant; "other" includes patients who died before post-induction could be delivered or who received off-trial therapy; (5) Genetic subtype: Standard/Low risk patients with those with other genetic abnormalities not classified into one of the other categories. High-risk - patients with any *KMT2A* rearranagement; Very high risk - low hypodiploidy/near-triploidy, complex karyotype or JAK-STAT abnormalities; Tyrosine kinase senstivie - *BCR-ABL1* fusion and ABL-class fusions. (6) hazard ratio respresents the risk of an event for patients with that specific copy number alteration compared with all other tested cases; (7) Hazard ratios for EFS, RR and OS were not materially altered when analysis was restricted to patients receving an allo-SCT.

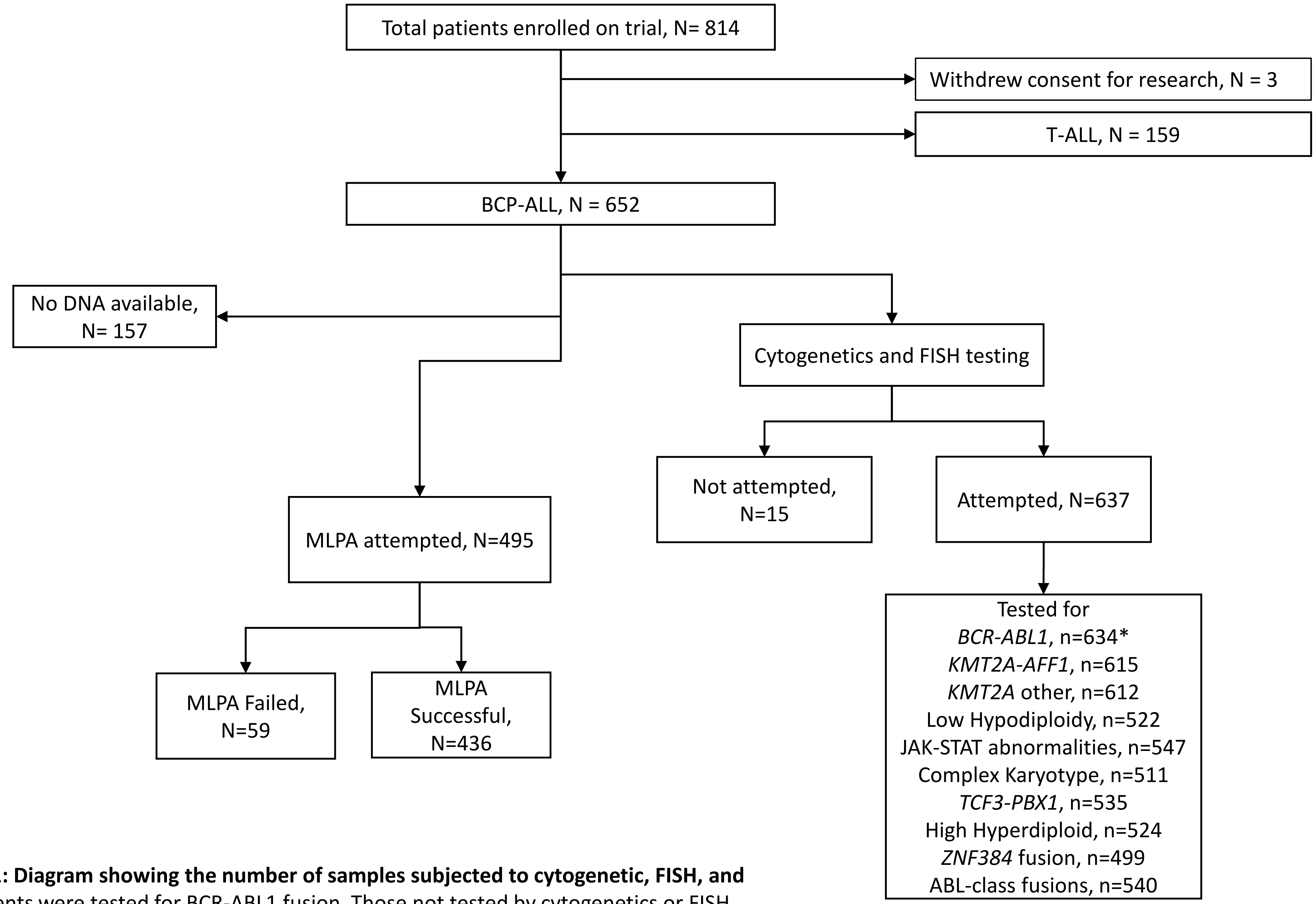

Supplementary Figure 1: Diagram showing the number of samples subjected to cytogenetic, FISH, and MLPA testing. * All patients were tested for BCR-ABL1 fusion. Those not tested by cytogenetics or FISH were screened by RT-PCR.

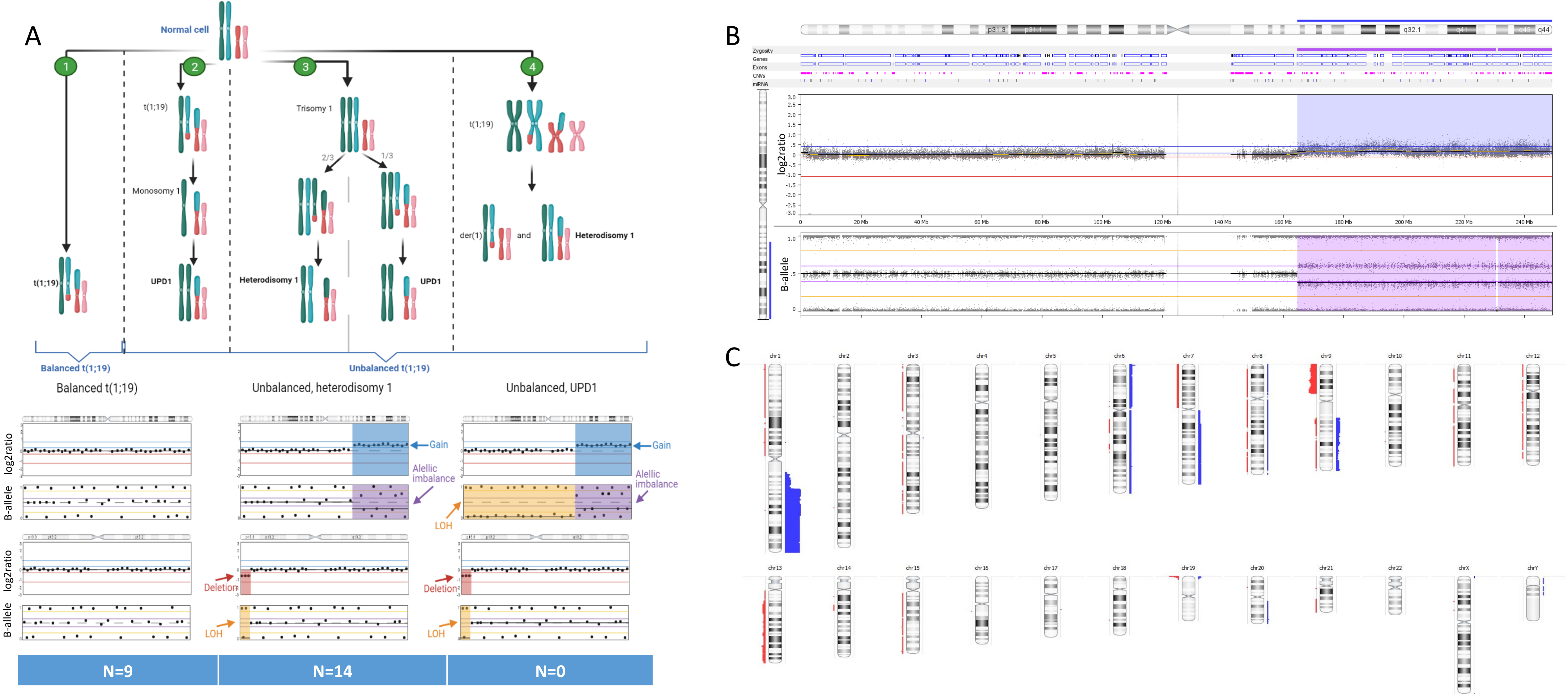

Supplementary Figure 2: Results of SNP analysis on 23 patients with t(1;19)(q21;p13) /*TCF3-PBX1* from UKALL14 (n=14) or UKALLXII (n=9). (A) The top panel outlines the potential mechanisms for the formation of t(1;19). The middle panel summarises the SNP profile for chromosome 1 expected in each scenario. The table shows the distribution of the 23 cases. Mechanism 2 and 3 would result in 100% and 66% cases respectively having UPD1. These results rule out mechanism 2. Assuming that the generation of heterodisomy 1 and UPD1 via mechanism 3 is random, the probability of seeing a ratio of 14:0 is <0.0001 (X^2 test); (B) Screenshot from NEXUS Copy Number Software showing an example of heterodisomy of chromosome 1; (C) Overview of the spectrum of copy number alterations observed in this cohort of t(1;19) cases. Blue lines to the right of each chromosome indicate gains while red lines to the left of each chromosome indicate deletions.

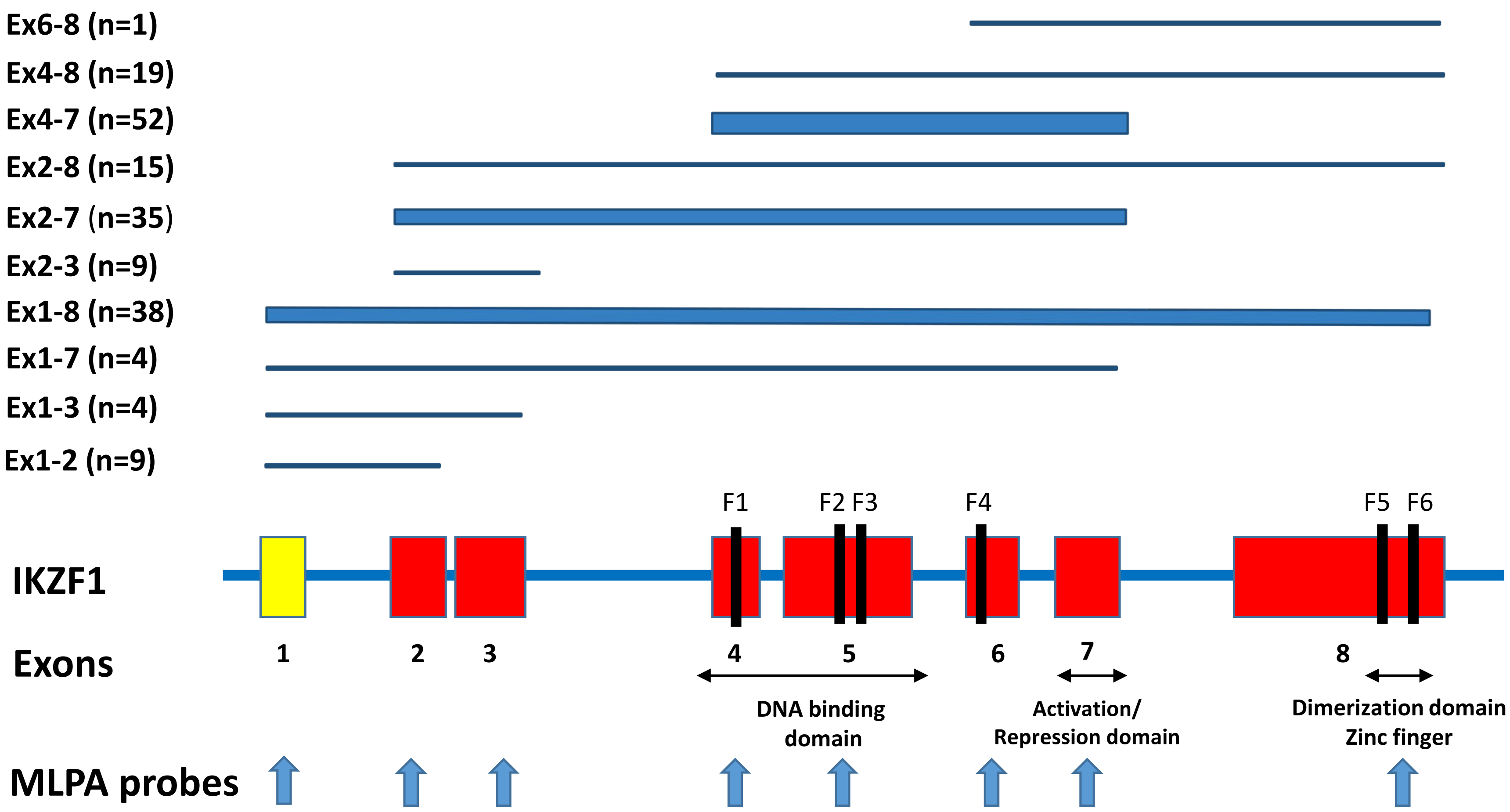

Supplementary Figure 3: Schematic diagram showing the type and distribution of IKZF1 deletion in Adult ALL. A total of 189 deletions were identified in 170 patients with 19 patients having two deletions. Co-occurring deletions were: Ex2-7/Ex2-3 (n=4); Ex4-8/Ex4-7 (n=3); Ex2-7/Ex4-7 (n=3); Ex1-8/Ex2-7 (n=2); Ex2-8/Ex 4-7 (n=2); Ex4-7/Ex2-3 (n=1); Ex1-8/Ex4-7(n=1); Ex2-8/Ex2-7 (n=1); Ex4-8/Ex 1-2 (n=1);Ex6-8/Ex 1-2 (n=1). Three patients with complex deletions are not included in the figure.

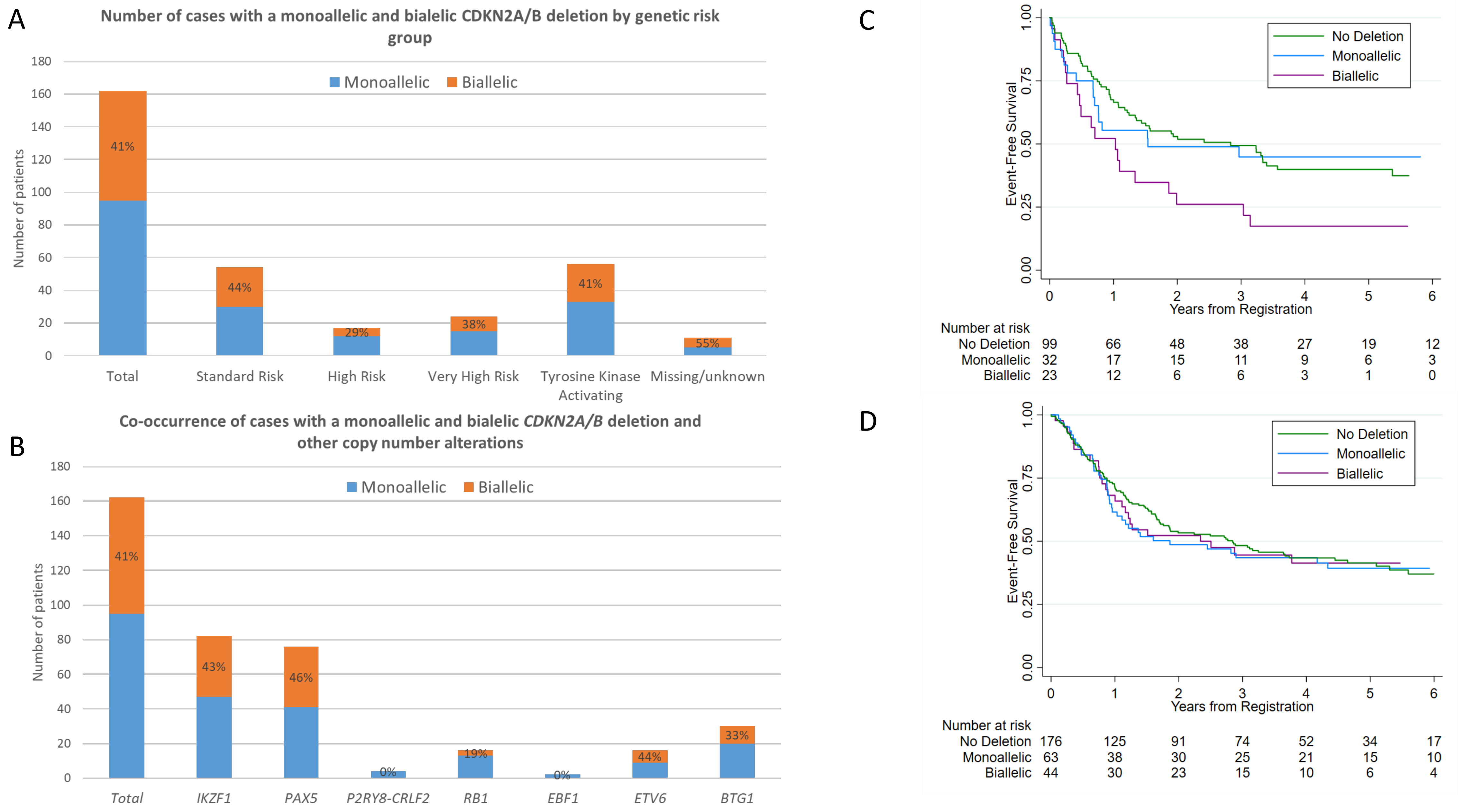

Supplementary Figure 4: Frequency and prognostic impact of monoallelic and biallelic *CDKN2A/B* deletions in adult B-cell precursor ALL. Biallelic *CDKN2A/B* deletions were defined as any *CDKN2A/B* deletion where the probe ratio for at least two of the three probes covering the *CDKN2A/B* locus were <0.25. All other deletions were classified as monoallelic deletions. (A + B) Bar charts showing the percentage of *CDKN2A/B* deletions that are biallelic according to the revised UKALL genetic risk group and by presence of other copy number alterations. (C & D) Kaplan survival graph showing the event free survival of *BCR-ABL1* positive (C) and negative (D) cases by *CDKN2A/B* copy number status

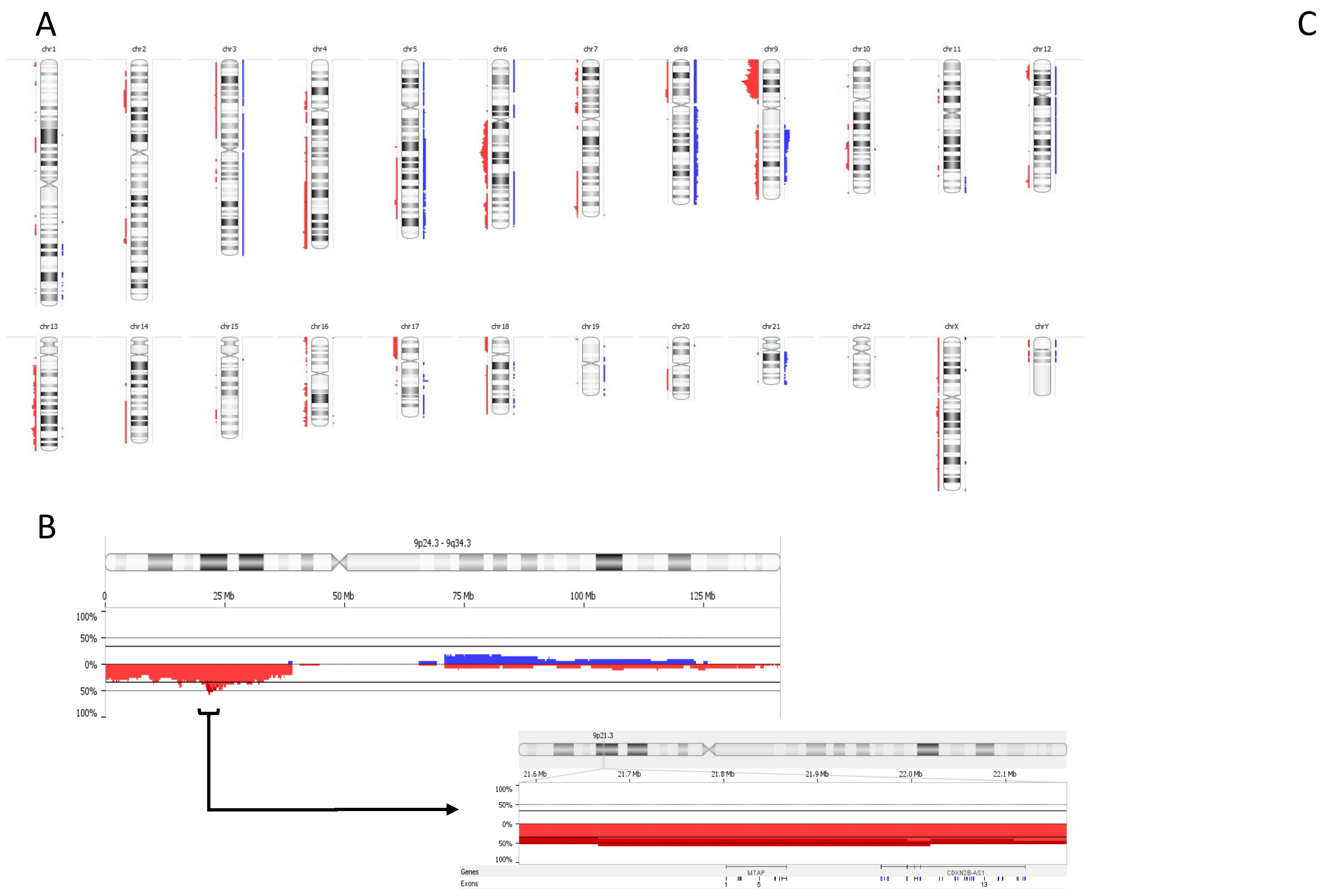

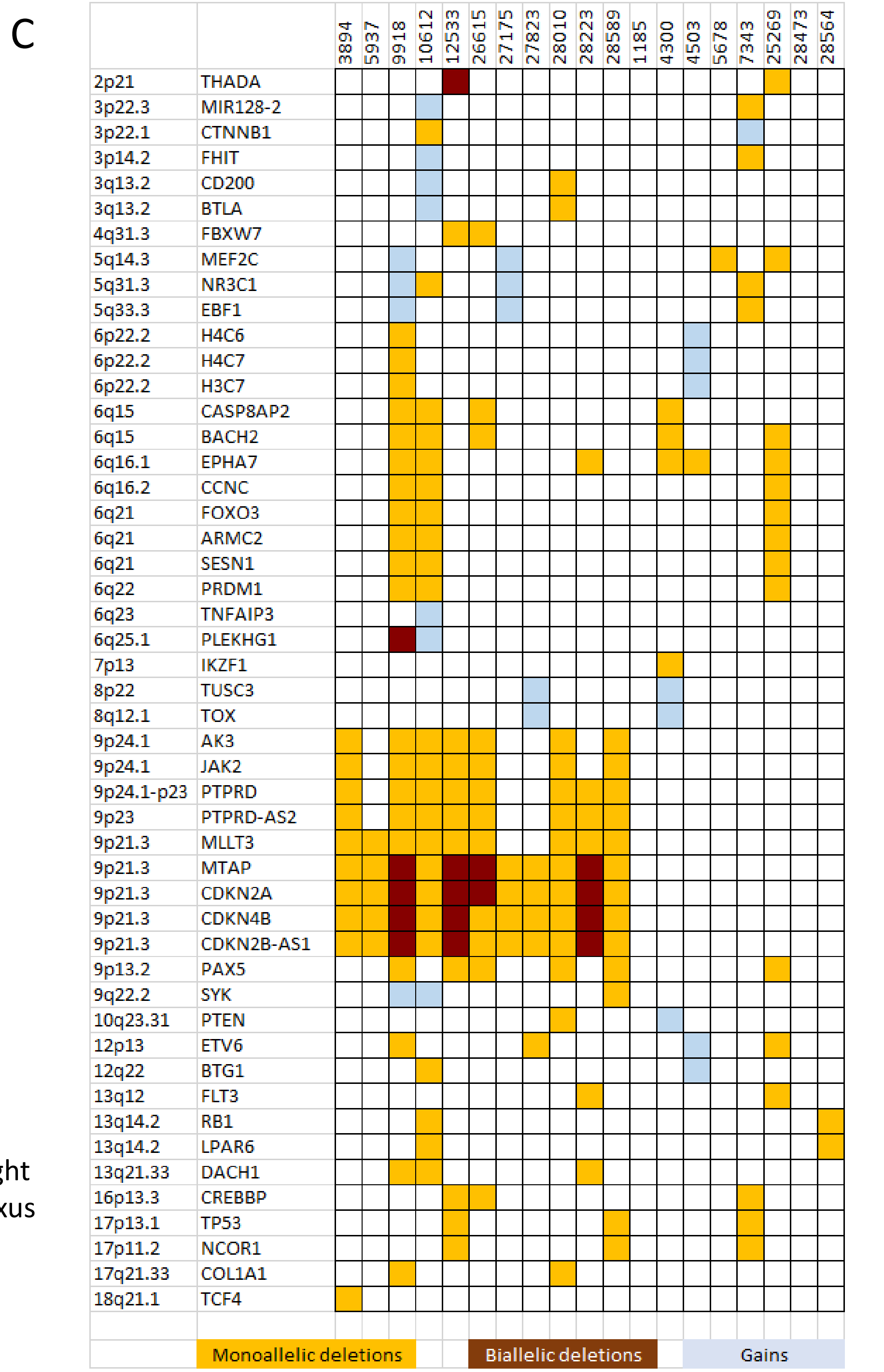

Supplementary Figure 5: Results of SNP analysis on 19 patients with B-cell precursor ALL and a complex karyotype from UKALL14 (n=9) or UKALLXII (n=10). (A) Overview of the spectrum of copy number alterations observed. Blue lines to the right of each chromosome indicate gains while red lines to the left of each chromosome indicate deletions; (B) Ideogram and Nexus screenshot illustrating the architecture of 9p deletions. All 9p deletions extended beyond *CDKN2A/B* to include *MTAP; (C)* Oncoplot detailing the copy number state of selected ALL relevant genes across the genome amongst cases with complex karyotype. A total of 11/19 (58%) cases had 9p deletions encompassing *CDKN2A*, *CDKN2B* and *MTAP.* Seven out of 19 cases (37%) harboured 6q deletions with *BACH2* (n=5) and *EPHA7* (n=7) being the most frequently deleted genes.
